# Impact of SGLT2 inhibition on markers of reverse cardiac remodelling in heart failure: Systematic review and meta‐analysis

**DOI:** 10.1002/ehf2.14993

**Published:** 2024-07-26

**Authors:** Patrick Savage, Chris Watson, Jaimie Coburn, Brian Cox, Michael Shahmohammadi, David Grieve, Lana Dixon

**Affiliations:** ^1^ Wellcome‐Wolfson Institute for Experimental Medicine Queen's University Belfast Belfast UK; ^2^ Royal Victoria Hospital Belfast UK

**Keywords:** heart failure, SGLT2 inhibitors, cardiac imaging, remodelling, biomarkers

## Abstract

**Introduction:**

Several landmark randomized‐controlled trials (RCTs) have demonstrated the efficacy of sodium‐glucose co‐transport 2 (SGLT2) inhibitors in reducing all‐cause mortality, cardiovascular (CV) mortality and heart failure (HF) hospitalizations. Much interest surrounds their mechanism of action and whether they have direct effects on reverse cardiac remodelling. Therefore, we conducted a meta‐analysis of placebo controlled RCTs evaluating the impact of SGLT2 inhibition on imaging derived markers of reverse cardiac remodelling in patients with HF.

**Methods:**

We performed a systematic review and meta‐analysis in accordance with the Preferred Reporting Items for Systematic Review and Meta‐Analysis (PRISMA) Statement and Cochrane Collaboration. Data interrogation of each major database including PubMed, EMBASE, MEDLINE and Cochrane Library was performed.

RCTs evaluating HF patients >18 years comparing SGLT2 inhibitor versus placebo‐control were included. Outcome measures included left ventricular end‐diastolic volume and volume index (LVEDV/LVEDVi), left ventricular end‐systolic volume and volume index (LVSDV/LVSDVi), left ventricular ejection fraction (LVEF), left ventricular mass index (LVMi), left atrial volume index (LAVi) and left ventricular global longitudinal strain (LV GLS). Studies with an HF with preserved ejection fraction population were excluded from analysis of parameters, which would be significantly affected by baseline LVEF, such as volumes and LVEF. The mean difference and standard error were extracted from each study and a random effects model used pool the mean difference and standard error across studies. A pre‐specified sub‐group analysis was performed to stratify results according to imaging modality used (cardiac magnetic resonance imaging and echocardiography). This study is registered on PROSPERO: CRD42023482722.

**Results:**

Seven randomized, placebo‐controlled trials in patients with HF comprising a total population of 657 patients were included. Overall LVEF of included studies ranged from 29 ± 8.0% to 55.5 ± 4.2%. In studies included in analysis of HFrEF parameters, baseline LVEF ranged from 29 ± 8% to 45.5 ± 12%. Pooled data demonstrated SGLT2 inhibition, compared with placebo control, resulted in significant improvements in mean difference of LVEDV [−11.62 ml (95% confidence interval, CI −17.90 to −5.25; *z* = 3.67, *P* = 0.0004)], LVEDVi [−6.08 ml (95% CI −9.96 to −2.20; *z* = 3.07; *P* = 0.002)], LVESV [−12.47 ml (95% CI −19.12 to −5.82; *z* = 3.68; *P* = 0.0002)], LVESVi [−6.02 ml (95% CI −10.34 to −1.70; *z* = 2.73; *P* = 0.006)], LVM [−9.77 g (95% CI −17.65 to −1.89; *z* = 2.43; *P* = 0.02)], LVMi (−3.52 g [95% CI −7.04 to 0.01; *z* = 1.96; *P* = 0.05)] and LVEF [+2.54 mL (95% CI 1.10 to 3.98; *z* = 3.62; *P* = 0.0005)]. No significant difference in GLS (*n* = 327) [+0.42% (95%CI −0.19 to 1.02; *P* = 0.18)] or LAVi [−3.25 ml (95% CI −8.20 to 1.69; *z* = 1.29; *P* = 0.20)] was noted.

**Conclusion:**

This meta‐analysis provides additional data and insight into the effects of SGLT2 inhibition on reverse cardiac remodelling in patients with HF. Compared with placebo control, we found that treatment with a SGLT2 inhibitor produced significant improvements in several markers of reverse cardiac remodelling.

## Introduction

Several landmark randomized‐controlled trials (RCT)s have demonstrated the efficacy of sodium‐glucose co‐transport 2 (SGLT2) inhibitors in reducing all‐cause mortality, cardiovascular (CV) mortality and rates of heart failure (HF) hospitalizations in patients with HF.^1^, ^2^, ^3^, ^4^, ^5^ This effect appears to be independent to anti‐diabetic effects and extends across the spectrum of left ventricular (LV) dysfunction. This has led to their rapid incorporation into international HF guidelines for HF with both reduced (HFreF) and preserved ejection fraction (HFpEF).^6^, ^7^ However, many questions remain unanswered as to their precise mechanisms of action in HF, with several suggested hypotheses such as modulation of inflammatory and metabolic pathways, as well as augmentation of myocardial energetics.^8^, ^9^

Much interest surrounds the mode of action and whether this pertains to synergistic systemic effects or those of direct cardiac action. Several RCT's have evaluated the direct impact of SGLT2 inhibitors on cardiac structure and function using cardiac imaging modalities such as magnetic resonance imaging (MRI) and echocardiography.^10^, ^11^ Although these trials have shown positive effects on markers of cardiac remodelling, many of these early studies have been confined to diabetic patients without HF and thus conclusions derived from these datasets may not be applicable to non‐diabetic patients with HF.

More recently, several RCT's enrolling non‐diabetics with HF have evaluated the direct effects of SGLT2 inhibition using cardiac imaging modalities.^10^, ^11^, ^12^, ^13^, ^14^ To our knowledge, no meta‐analysis of placebo controlled RCT's evaluating the impact of SGLT2 inhibition on markers of cardiac remodelling in patients with HF (including a non‐diabetic population) have been performed. We therefore conducted a systematic review and meta‐analysis of patients with HF to evaluate the impact of SGLT2 inhibition on markers of cardiac remodelling. We hypothesized that SGLT2 inhibition would be associated with favourable changes in markers of cardiac remodelling in patients with HF.

## Methods

### Data search strategy

We performed systematic review and meta‐analysis in accordance with the Preferred Reporting Items for Systematic Review and Meta‐Analysis (PRISMA) Statement and Cochrane Collaboration. The literature search was designed by an information specialist utilizing selected medical subject heading (MeSH) terms and free‐text terms associated with SGLT2 inhibitors and LV remodelling. Data interrogation of each major database including PubMed, EMBASE, MEDLINE and Cochrane Library was performed with screening of references of the included studies, reviews and meta‐analyses to identify any additional trials.

**Figure 1:**
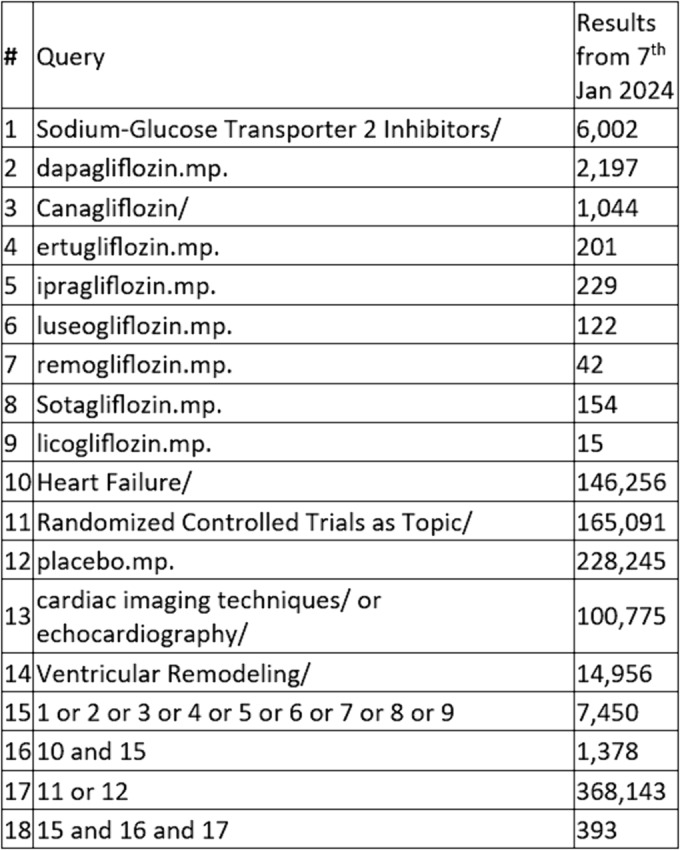
Search strategy for data extraction from EMBASE. A full detailed strategy for each database is available in the supplementary material.

A data search was performed from inception of SGLT2 inhibitors up to and including 7 January 2024. Studies comparing the effects of SGLT2 inhibitors (empagliflozin, canagliflozin, dapagliflozin, ertugliflozin, ipragliflozin, luseogliflozin, remogliflozin, sotagliflozin and licogliflozin) on cardiac remodelling parameters, evaluated using echocardiography or cardiac magnetic resonance imaging in patients with HF were included. A detailed search strategy is shown in Figure 1 with full strategies for each database provided in the supporting information (Appendix sect. 2.0). We included only randomized controlled trials published in English. Observational and cohort studies were excluded. Case reports, series, editorial, letters and perspectives were also excluded. This study has been registered at PROSPERO with registration number CRD42023482722.

### Screening criteria

Screening and study selection was performed according to a PICOS framework with full details outlined **in** Table 1. In brief, we included randomized controlled trials comparing the effects of SGLT2 inhibition vs placebo on markers of left ventricular remodelling as evaluated using cardiac imaging with either echocardiography or cardiac magnetic resonance imaging. Populations included were patients >18 years of age, of both male and female gender, diagnosed with either HF with reduced ejection fraction (HFrEF) or HF with preserved ejection fraction (HFpEF). Outcome measures included for analysis included, left ventricular end‐diastolic volume and volume index [LVEDV (mL)/LVEDVi (mL/m^2^)], left ventricular end‐systolic volume and volume index [LVSDV (mL) /LVSDVi (mL/m^2^)], left ventricular ejection fraction (LVEF) (%), left ventricular mass (LVM) index (LVMi) (g/m^2^), left atrial volume [LAV (mL)] and left atrial volume index [LAVi (mL/m^2^)] and left ventricular global longitudinal strain (%). Full screening and study selection was performed according to a PICOS framework (Table 1).

**Table 1:** PICOS table.

PICOS	Inclusion	Exclusion criteria
Population	Patients with heart failure	
Intervention	SGLT2 inhibitors, including empagliflozin, canagliflozin, dapagliflozin, ertugliflozin, ipragliflozin, luseogliflozin, remogliflozin, sotagliflozin and licogliflozin.	
Comparison	Effects of SGLT2 inhibition vs. placebo on left ventricular remodelling evaluated using cardiac imaging (echocardiography or cardiac magnetic resonance imaging).	
Outcome	Left ventricular end‐diastolic volume [LVEDV (mL)]	
	Left ventricular end‐systolic volume [LVESV (mL)]	
	Left ventricular mass [LVM (g)]	
	Left ventricular end‐diastolic volume and volume index [LVEDVi (mL/m^2^)]	
	Left ventricular end‐systolic volume and volume index [LVSDVi (mL/m^2^)]	
	Left ventricular ejection fraction [LVEF) (%)]	
	Left ventricular mass index [LVMi] (g/m^2^)]	
	Left atrial volume index [LAVi (mL/m^2^)]	
	Left ventricular global longitudinal strain (%).	
Study design	Randomized controlled trials	Case studies
	Databases including PubMed, Embase, Cochrane & MEDLINE.	Cohort studies
		Editorials, letters and perspectives

Abbreviations: LA, left atrial; LVEDV, left ventricular end‐diastolic volume; LVEDVi, left ventricular end‐diastolic volume index; LVEF, left ventricular ejection fraction; LVESV, left ventricular end‐systolic volume; LVESVi, left ventricular end‐systolic volume index; LVGLS, left ventricular global longitudinal strain; LVM, left ventricular mass; LVMi, left ventricular mass index; PICOS, Populations, Intervention, Comparison, Outcomes and Study; SGLT2, sodium‐glucose cotransporter 2.

### Study selection and data extraction

**Figure 2:**
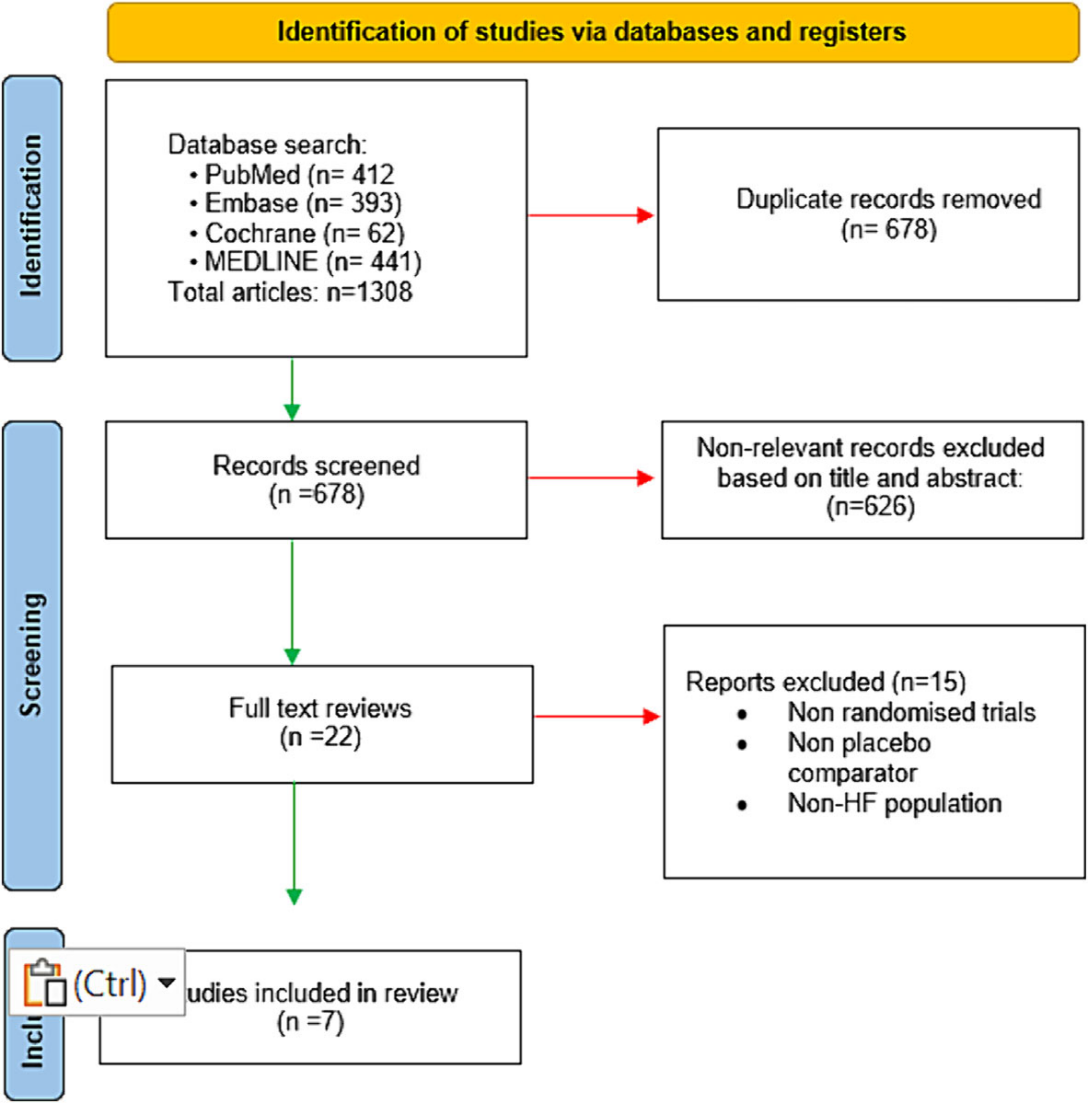
PRISMA Flow chart detailing identification and processing of article selection.

After the initial literature search utilizing MeSh terms relating to SGLT2 inhibitors and LV remodelling from relevant databases, two independent reviewers (B. C. and M. S.) independently screened titles and abstracts for eligible studies according to the inclusion and exclusion criteria as detailed in Figure 2. A third author (P S.) acted to resolve any discrepancies in eligibility. Data extraction was then performed by B. C. and M. S. detailing general information, population and settings, methods, participants, interventions, outcomes and results.

### Risk of bias assessment

The risk of bias of the included studies was assessed by a fourth reviewer (J. C.) using a detailed framework provided by the Cochrane Handbook for Systematic Reviews of Interventions. This tool includes six domains: random sequence generation, allocation concealment, blinding, incomplete outcome data, selective outcome reporting and other bias. The six domains were separately evaluated and categorized as low, unclear or high risk of bias.

### Data analysis

The mean difference (MD) and standard error were extracted from each study. A random effects model was used to pool the mean difference and standard error across studies.^15^ Heterogeneity between studies was assessed using the *Q* statistic, and its extent was calculated by the *I* ^2^ test; values <25% indicated low heterogeneity, whereas values between 26% and 75% reflected moderate heterogeneity and 76% to 100% indicating high.^16^ A pre‐specified sub‐group analysis was performed to stratify results according to imaging modality used (cardiac MRI and echocardiography). Funnel plot asymmetry was visually assessed to look for evidence of potential publication bias with Begg's and Egger's test utilized for quantitative assessment. Sensitivity analysis was planned, restricting analysis to studies assessed to have low risk of bias (following assessment of risk of bias utilizing the framework detailed in the Cochrane handbook). In addition, given differing imaging modalities used, robustness of effect was further assessed with a repeat analysis utilizing standardized mean difference (SMD). In the case of missing data relevant to the analysis was encountered, efforts to obtain relevant data from the corresponding author of the respective paper were pursued. Statistical analysis was performed using Review Manager (RevMan) Version 5.4^17^ and JASP Version 0.18.3.^18^

## Results

### Included studies

A literature search of four databases (PUBMED, MEDLINE, COCHRANE and EMBASE) retrieved 1308 results. After screening for duplication and non‐relevant titles, full text analysis of 22 articles were performed with seven clinical trials included in the final analysis. A PRISMA flowchart is shown in Figure 2. The seven final included studies were randomized, placebo‐controlled trials in patients with HF, comprising a total population of 657 patients, 327 (49.8%) of whom were treated with an SGLT2 inhibitor. Four studies used empagliflozin (10‐mg OD) with three studies using dapagliflozin (10‐mg OD) (Table 2). The mean age ranged from 62 to 70.5 years of age with the majority of patients being male 484 (73.7%). In assessment of remodelling parameters specific to HFrEF [LVEDV, LVEDVi, left ventricular end‐systolic volume (LVESV), LVESV index (LVESVi) and LVEF] baseline LVEF in included studies ranged from 29 ± 8% to 45.5 ± 12%. Patients with HFpEF comprised 5.5% (*n* = 36) of the total population. Full demographics are listed in (Table 3). Baseline HF therapies were similar between groups and are detailed in Table 4. Follow‐up ranged from 12 weeks to 1 year, and imaging modalities employed were either cardiac MRI (four studies) or echocardiography (three studies). Of note, data from EMPA‐VISION are presented as separate HFrEF and HFpEF cohorts, given these data were analysed separately in the original study.

**Table 2:** Final included studies.

Study	Registration No	Author	Year	Design	Follow‐up	Population	Imaging modality	Drug	Control	Total (*n*, %)	Treatment (*n*, %)	Control (*n*, %)
EMPATROPSIM	NCT03485222	Santos‐Gallego *et al*.	2021	RCT	6 months	HF	CMRI	Empagliflozin 10‐mg OD	Placebo	84 (100)	42 (50)	42 (50)
EMPA‐VISION	NCT03332212	Hundertmark *et al*.	2023	RCT	12 weeks	HF	CMRI	Empagliflozin 10‐mg OD	Placebo	72 (100)	35 (48.6)	37 (51.4)
SUGAR‐DM	NCT03485092	Lee *et al*.	2020	RCT	36 weeks	HF	CMRI	Empagliflozin 10‐mg OD	Placebo	105 (100)	52 (49.5)	53 (50.5)
REFORM	NCT02397421	Singh *et al*.	2020	RCT	12 months	HF	CMRI	Dapagliflozin 10‐mg OD	Placebo	56 (100)	28 (50)	28 (50)
EMPIRE HF	NCT03198585	Omar *et al*.	2021	RCT	12 weeks	HF	Echocardiography	Empagliflozin 10‐mg OD	Placebo	190 (100)	95 (50)	95 (50)
Qianyu Fu et al.	**ChiCTR**2300072707	Qianyu Fu *et al*.	2023	RCT	12 months	HF	Echocardiography	Dapagliflozin 10‐mg OD	Placebo	60 (100)	30 (50)	30 (50)
DAPA‐VO2	NCT04197635	Palau *et al*.	2022	RCT	3 months	HF	Echocardiography	Dapagliflozin 10‐mg OD	Placebo	90 (100)	45 (50)	45 (50)

*Note*: The table demonstrates the seven final trials included in analysis. Trial registration data, lead author, duration of treatment and SGLT2 inhibitor utilized are noted in addition to imaging modality used. Numbers are presented as (*n*, %).

**Table 3:** Demographics of final included studies.

Study details	Demographics			Comorbidities			Classification HF (*n*, %)			NYHA class (*n*, %)				Baseline LVEF (%)
	Mean age (mean ± *SD*)	Male (*n*, %)	Female (*n*, %)	Hypertension (*n*, %)	IHD (*n*, %)	BMI (kg/m2)	Diabetes	Ischaemic	Non‐ischaemic	I	II	III	IV	
EMPATROPSIM	62 ± 12.1	54 (64%)	46 (27)	62 (74)	42(50)	29.7	0 (0)	42 (50)	41 (49)	NR	NR	NR	NR	36 ± 8
EMPA‐VISION	68.33 ± 11.51	42 (58.3%)	30 (41.7%)	20 (27.8)	NR	30.1	9 (12.5)	NR	NR	0	59 (81.9)	12 (16.7)	0 (0)	HFrEF ‐ 38.1 ± 9.5
						30.7^a^								HFpEF‐ 55.5 ± 4.2*
SUGAR‐DM	68.7 ± 11.1	77 (73.3)	28 (26.7%)	74 (70.5)	74 (70.5)	30.7	82 (78.1)	NR	NR	0	81 (77.1)	24 (22.9)	1 (1.4%)	32.5 ± 9.8
REFORM	67.1 ± 6.9	37 (66.1%)	19 (33.9%)	40 (71.4)	29 (51.8)	32.5	56 (100)	30 (53.6)	26 (46.4)	25 (44.6)	24 (42.9)	7 (12.5)	NR	45.5 ± 12
EMPIRE HF	64 ± 11.0	162 (85.3%)	28 (14.7%)	76 (40)	103 (54.2)	29	24 (12.6)	97 (51.1)	93 (48.9)	12	149	29	0	29 ± 8
*Qianyu Fu et al*.	70.5 ± 6.4	43 (71.7%)	17 (28.3%)	NR	NR	24.7	60 (100)	32 (53.4)	21 (35)	NR	41 (68.4)	16 (26.7)	3(5)	30.9 ± 3.7
DAPA‐VO2	68.6 (61.5–74.2)	69 (76.7)	21 (23.3%)	70 (77.8)	71 (78.8)	27.7	29 (32.2)	NR	NR	NR	80 (88.9)			33.8 ± 5.3

*Note*: The table displays the demographics of the seven included studies in final analysis. Proportions are presented as (*n*, %) with age and LVEF presented as mean ± *SD*.

Abbreviations: BMI, body mass index; HF, heart failure; IHD, ischaemic heart disease; LVEF, left ventricular ejection fraction NYHA, New York Heart Classification; *SD*, standard deviation.

^a^ Mean LVEF ± *SD* presented for the HFrEF and HFpEF populations which were analysed separately in EMPA‐VISION. Mean BMI presented separately for this population also.

**Table 4:** Baseline HF medication in final included studies.

Baseline heart failure medication															
Study details	Drugs placebo groups (*n*, %)					Drugs SGLT2 group (*n*, %)					Drugs combined (*n*, %)				
	Loop	ACEi/ARB	B‐blockers	MRA	ARNI	Loop	ACEi/ARB	B‐blockers	MRA	ARNI	Loop	ACEi/ARB	B‐blockers	MRA	ARNI
EMPATROPSIM	24 (57)	19 (45)	38 (90)	15 (36)	15 (36)	22 (52)	16 (38)	36 (86)	13 (31)	21 (50)	46 (55)	35 (42)	74 (88)	28 (33)	36 (43)
EMPIRE HF	62 (65)	65 (68)	89 (94)	63 (66)	27 (28)	63 (66)	59 (62)	91 (96)	62 (65)	31 (33)	125 (65.8)	124 (65.2)	180 (94.7)	125 (65.8)	58 (31.1)
EMPA‐VISION (HFrEF)	9 (47.4)	16 (84.2)	19 (100)	12 (63.2)	3 (15.8)	9 (52.9)	12 (70.6)	14 (82.3)	14 (82.3)	4 (23.5)	18 (47.4)	28 (73.7)	33 (86.8)	26 (68.4)	7 (18.4)
EMPA‐VISION (HFpEF)	12 (66.7)	5 (27.8)	6 (33.3)	5 (27.8)	0 (0)	11 (61.1)	9 (50)	14 (77.8)	3 (16.7)	0 (0)	23 (63.9)	14 (38.9)	20 (55.6)	8 (22.2)	0 (0)
SUGAR‐DM	29 (54.7)	36 (67.9)	50 (94.3)	31 (58.5)	15 (28.3)	31 (59.6)	28 (53.9)	46 (88.5)	32 (61.5)	21 (40.4)	60 (57.1)	64 (61)	96 (91.4)	63 (60)	36 (34.3)
REFORM	NR	25 (89.3)	22 (78.6)	10 (35.7)	NR*	52.9 (20.5)	25 (89.3)	24 (85.7)	13 (46.4)	NR*	NR	50 (89.3)	46 (82.1)	23 (41.1)	NR*
ChiCTR2300072707	28 (93.3)	7 (23.3)	26 (86.7)	22 (73.3)	22 (73.3)	27 (90)	7 (23.4)	25 (83.3)	20 (66.7)	20 (66.7)	55 (91.7)	14 (23.3)	51 (85)	42 (70)	27 (45)
DAPA‐VO2	38 (84.4)	3 (6)	41 (91.1)	32 (71.1.)	40 (88.9)	39 (86.7)	4 (8)	41 (91.1)	32 (71.1)	44 (97.8)	77 (85.6)	7 (7)	82 (91.1)	67 (74.4)	80 (88.9)

*Note:* The table shows the baseline heart failure medication of final included studies. Proportions are presented as (*n*, %).

Abbreviations: ACEi, angiotensin‐converting enzyme inhibitor; ARB, angiotensin receptor blocker; ARNI, angiotensin–neprilysin inhibitor; MRA, mineralocorticoid receptor antagonist; NR, not recorded.

### Risk of bias assessment

A risk of bias assessment of each randomized clinical trial was performed by an independent reviewer (J. C.) using a dedicated Cochrane ROB assessment tool. Overall risk of bias in each of the studies included was low. Full summary risk of bias graphs and tables are included in Appendix sect. 3.0 (Appendix 3.0: Figures S1 and S2). Publication bias was tested visually using funnel plots and quantitatively with both Begg's and Egger's test; *P* values <0.05 were considered statistically significant (Appendix 4.0: Figures S3–S11).

### LVEDV and LVEDVi

**Figure 3:**
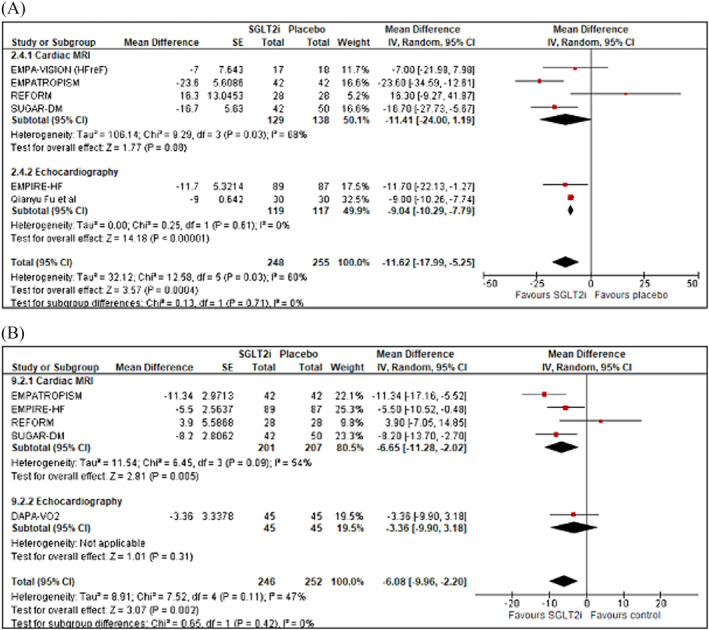
Forrest plot demonstrating mean difference in (A) LVEDV (mL) and (B) LVEDVi (mL/m^2^) from baseline to study end in RCTs of patients with heart failure treated with SGLT2 inhibitor therapy versus controls. CI, confidence interval; LVEDV, left ventricular end‐diastolic volume; LVEDVi, left ventricular end‐diastolic volume index; RCT, randomized controlled trial; SE, standard error.

Six studies assessed LVEDV (mL) (*n* = 503) and five assessed LVEDVi (mL/m^2^) (*n* = 498) (Figure 3). Pooled data from studies evaluating LVEDV demonstrated that SGLT2 inhibition, compared with controls, significantly decreased LVEDV by and overall mean difference of −11.62 mL (95% CI −17.99 to −5.25; *z* = 3.67, *P* = 0.0004). A similar improvement was seen for LVEDV indexed to BSA [(LVEDVi (mL/m^2^)], with an observed mean difference of −6.08 mL (95% CI −9.96 to −2.20; *z* = 3.07; *P* = 0.002). In a pre‐specified sub‐group analysis between imaging modality used, there was no difference between groups in change in LVEDV (CMR vs. echocardiography; *χ* ^2^ = 0.13, *df* = 1, *P* = 0.71, *I* ^2^ = 0%) or LVEDVi (CMR vs. echocardiography; *χ* ^2^ = 0.65, *df* = 1, *P* = 0.42, *I* ^2^ = 0%). No significant publication bias was noted with respect to LEDV; however, for LVEDVi, a degree of bias detected using Egger's test (Appendix 4.0: Figures S3, S4 and S10).

#### LVESV and LVESVi

**Figure 4:**
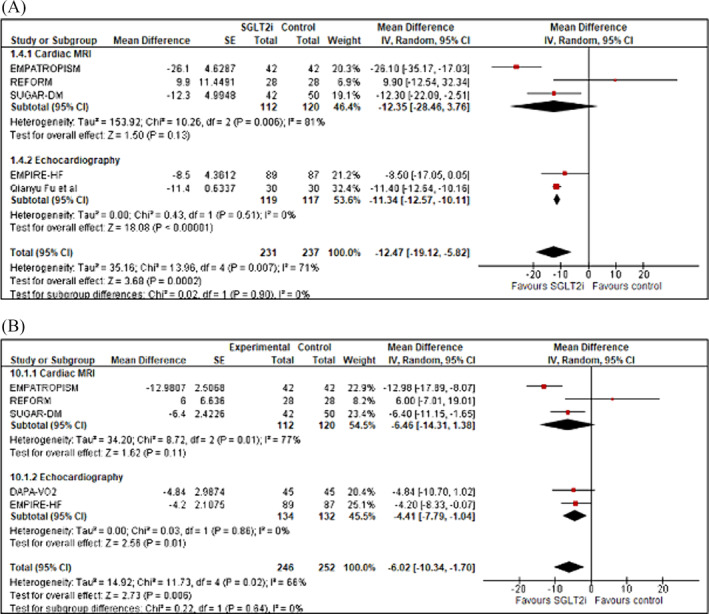
Forest plot demonstrating mean difference in (A) LVESV (mL) and (B) LVESVi (mL/m^2^) from baseline to study end in RCTs of patients with heart failure treated with SGLT2 inhibitor therapy versus controls. CI, confidence interval; LVESV, left ventricular end‐systolic volume; LVESVi, left ventricular end‐systolic volume index; RCT, randomized controlled trial; SE, standard error.

A total of five studies assessed LVESV (mL) (*n* = 468) and five assessed LVESVi (mL/m^2^) (*n* = 498) (Figure 4). Compared with control, LVESV was significantly reduced with SGLT2 inhibition with a pooled mean difference noted of −12.47 mL (95% CI −19.12 to −5.82; *z* = 3.68; *P* = 0.0002). In the studies that assessed LVESVi (mL/m^2^), a significant reduction following treatment with a SGLT2 inhibitor versus control was also noted [mean difference −6.02 mL (95% CI −10.34 to −1.70; *z* = 2.73; *P* = 0.006)]. There was no difference between imaging modalities used in either LVESV (CMR vs. echocardiography; *χ* ^2^ = 0.02, *df* = 1, *P* = 0.90, *I* ^2^ = 0%) or LVESVi (CMR vs. echocardiography; *χ* ^2^ = 0.22, *df* = 1, *P* = 0.64, *I* ^2^ = 0%). No significant publication bias was noted (Figures S3, S5 and S9).

### LVM and LVMi

**Figure 5:**
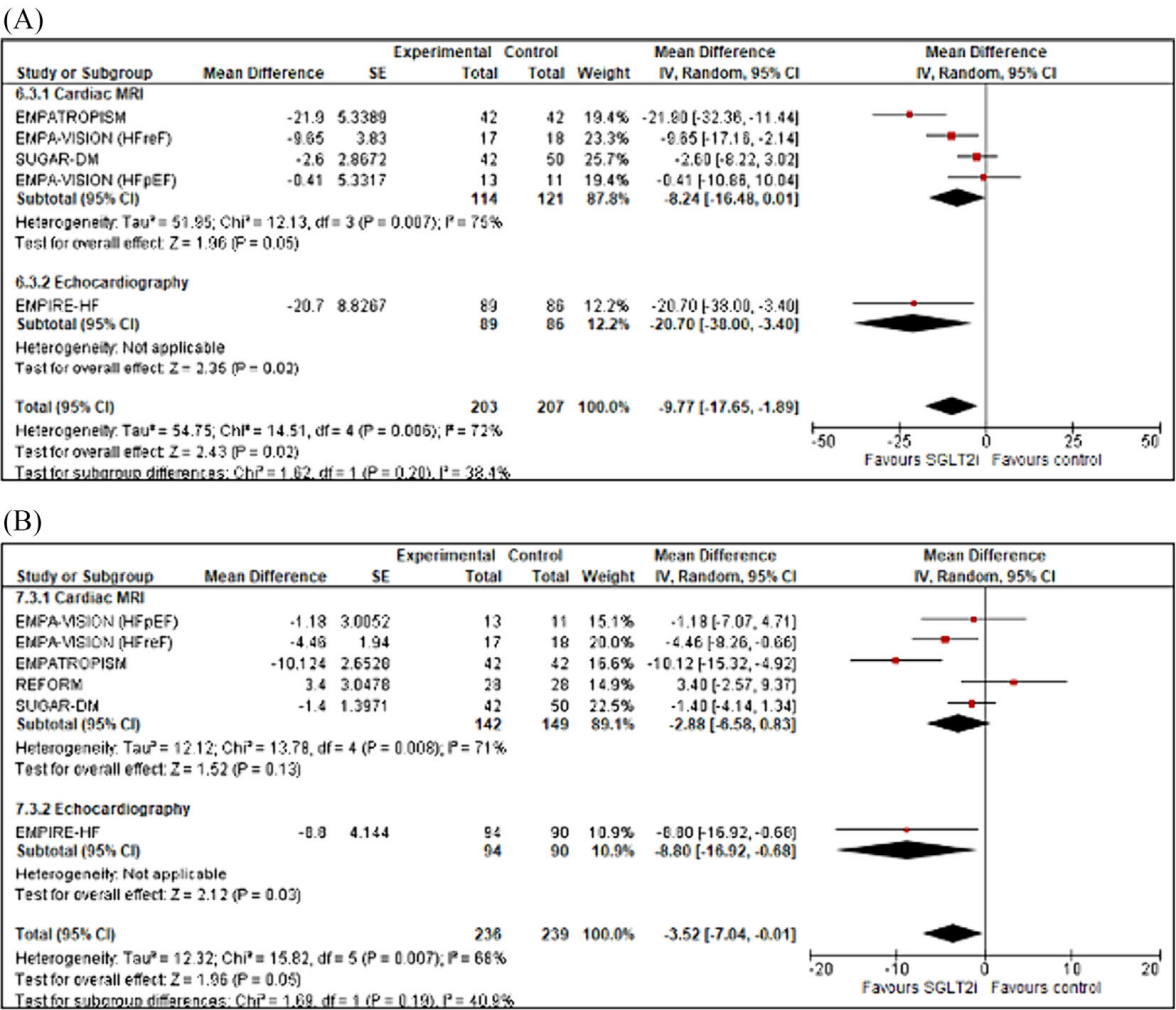
Forest plot demonstrating mean difference in (A) LVM (g) and (B) LVMi (g/m^2^) from baseline to study end in RCTs of patients with heart failure treated with SGLT2 inhibitor therapy versus controls. CI, confidence interval; RCT, randomized controlled trial; LVM, left ventricular mass; LVMi, left ventricular mass index; SE, standard error.

Four studies assessed LVM (*n* = 410) and five assessed LVMi (*n* = 475) (Figure 5). LVM was significantly reduced by SGLT2 inhibition versus control (mean difference −9.77 g [95% CI −17.65 to −1.89; *z* = 2.43; *P* = 0.02)] as was LVMi [mean difference −3.52 g [95% CI −7.04 to 0.01; *z* = 1.96; *P* = 0.05)]. There was no significant difference between imaging modality used for either LVM or LMVi (CMR vs. echocardiography; *χ* ^2^ = 1.62, *df* = 1, *P* = 0.20, *I* ^2^ = 38.4%) and (CMR vs. echocardiography; *χ* ^2^ = 1.39, *df* = 1, P = 0.19, *I* ^2^ = 40.9%) respectively. There was no evidence of publication bias (Appendix 4.0: Figures S3, S8 and S11).

### LAV index (mL/m^2^)

**Figure 6:**
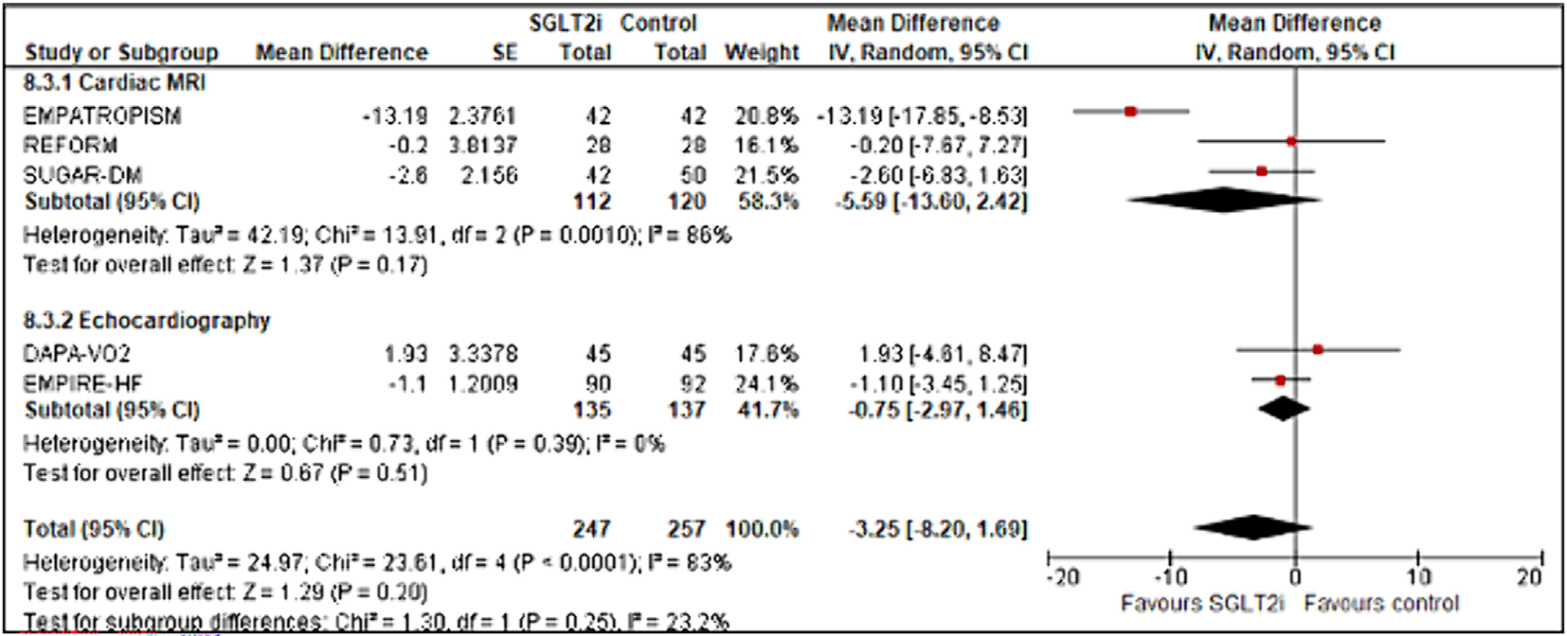
Forrest plot demonstrating mean difference in LAVi (mL/m^2^) from baseline to study end in RCTs of patients with heart failure treated with SGLT2 inhibitor therapy versus controls. CI, confidence interval; LAVi, left atrial volume index; RCT, randomized controlled trial; SE, standard error.

Five studies measured LAVi (*n* = 504) with a SGLT2 inhibition producing a pooled mean difference of −3.25 mL versus controls; however, this did not reach significance (95% CI −8.20 to 1.69; *z* = 1.29; *P* = 0.20) (Figure 6). In a sub‐group analysis of imaging modality used, no difference was noted between groups (*χ* ^2^ = 1.30, *df* = 1, *P* = 0.25, *I* ^2^ = 23.2%). No significant publication bias was noted. (Appendix 4.0: Figures S3 and S10).

### Left ventricular ejection fraction (%)

**Figure 7:**
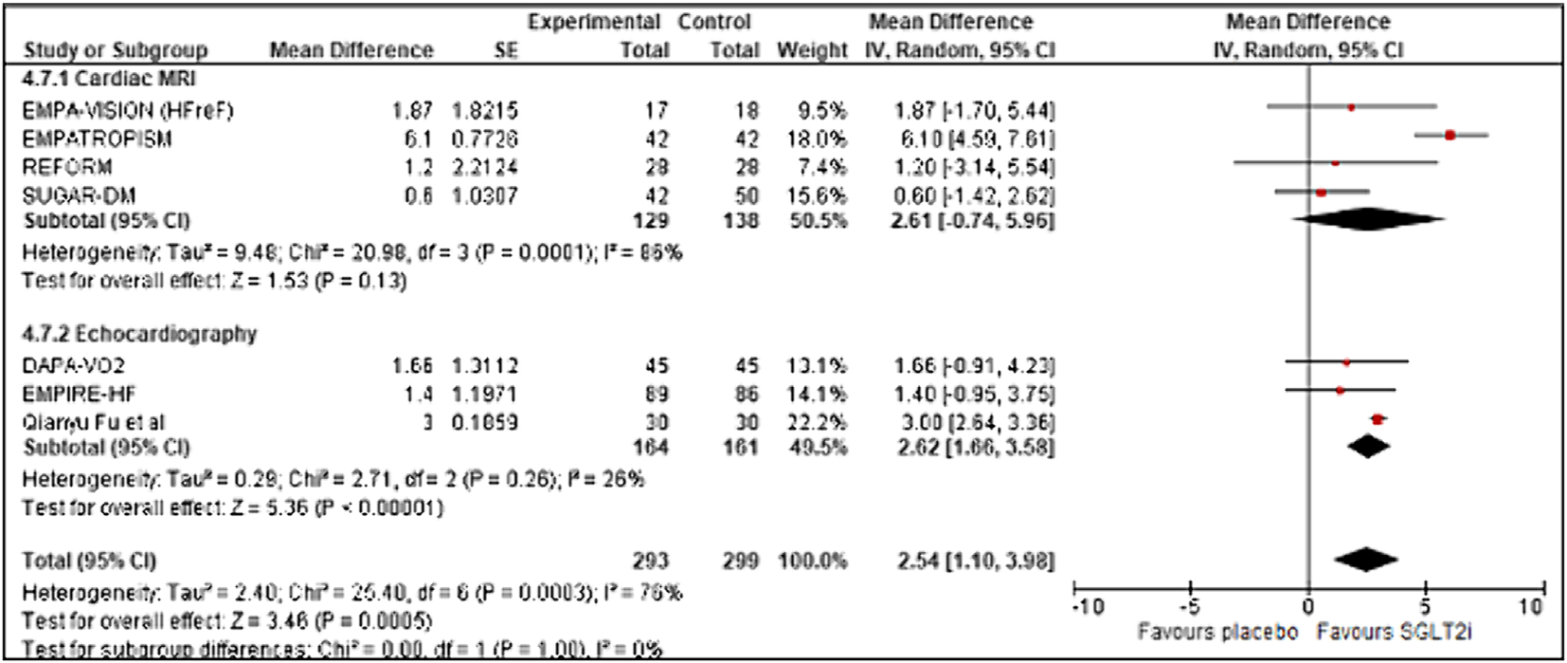
Forrest plot demonstrating mean difference in LVEF (%) from baseline to study end in RCTs of patients with heart failure treated with SGLT2 inhibitor therapy versus controls. CI, confidence interval; LVEF, Left ventricular ejection fraction; RCT, randomized controlled trial; SE, standard error.

Seven studies measured LVEF (*n* = 592) (Figure 7). A significant improvement in LVEF was noted with SGLT2 inhibition versus control with a mean increase of +2.54% (95% CI 1.10 to 3.98; *z* = 3.46; *P* = 0.0005). A high degree of heterogeneity was noted between studies (Tau^2^ = 2.20; *χ* ^2^ = 26.35, *df* = 7, *P* = 0.0004, *I* ^2^ = 73%). There was no difference between imaging modality used (*χ* ^2^ = 0.00, *df* = 1, *P* = 1.00, *I* ^2^ = 0%). No significant publication bias was noted (Appendix 4.0: Figures S3 and S6).

### Left ventricular global longitudinal strain (%)

**Figure 8:**
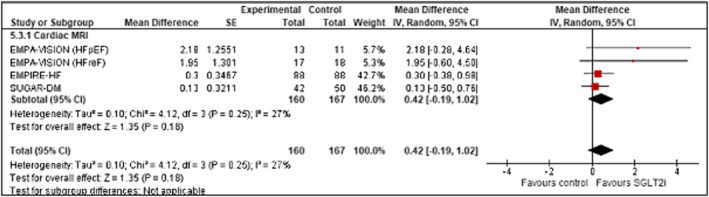
Forrest plot demonstrating mean difference in LV GLS (%) from baseline to study end in RCT's of patients with heart failure treated with SGLT2 inhibitor therapy versus controls. CI, confidence interval; RCT, randomized controlled trial; SE, standard error.

Three studies measured GLS (*n* = 327) (Figure 8). A trend towards improved LV GLS with SGLT2 inhibition versus controls was noted; however, this did not reach statistical significance (mean increase +0.42% [95% CI ‐0.19 to 1.02; *z* = 1.35; *P* = 0.18). No significant publication bias was noted (Appendix 4.0: Figures S3–S7).

### Sensitivity analysis

Sensitivity analysis excluding trials with high ROB was planned; however, risk of bias in the trials included was low and therefore precluded a secondary analysis based on this criterion alone. To further evaluate the robustness of effect, a secondary analysis was performed using a standardized mean difference (SMD) for LVEDV/LVEDVi/LVESV/LESVi/LVM/LMVi/LVEF and left ventricular global longitudinal strain (LV GLS). Similar effect sizes and directionality of results were noted with the overall results from our initial analysis remaining unchanged, therefore highlighting their robustness. Full results of this analysis are provided in the supporting information (Appendix 5.0).

A further analysis was performed with the exclusion of the REFORM study, due to its significant heterogeneity in outcomes with respect to LVESV, LVESVi, LVEDV, LVEDVi and LVMi (g/m^2^). After sensitivity analysis a greater improvement in volumetric parameters was noted (LVEDV, LVEDVi, LVESV & LVESVi). Heterogeneity was improved in LVEDV, LVEDVi and LVESVi. There was no difference noted in LVMi with overall treatment effect remaining the same. Full details of this analysis are included in Appendix 6.0.

## Discussion

This meta‐analysis of seven placebo‐controlled randomized trials provides additional data and insight into the effects of SGLT2 inhibition on reverse cardiac remodelling in patients with HF. We found that treatment with a SGLT2 inhibitor, compared with placebo, resulted in significant improvements in several markers of reverse cardiac remodelling, specifically, LVESV, LVESVi, LVEDV, LVEDVi, LVM, LVMi and LVEF. Previous meta‐analyses have been limited to studies evaluating diabetic patients, a large portion of which did not have HF.^19^ This analysis has pooled data from randomized trials, placebo‐controlled trials, enrolling HF patients with and without diabetes, adding valuable information to help understand the treatment effects of these drugs in HF.

Individually, only a small number of randomized studies have demonstrated significant changes in reverse cardiac remodelling following SGLT2 inhibition. This may be reflective of the often small sample sizes recruited. Indeed, of the individual studies in our analysis, only three demonstrated significant structural or functional changes.^10^, ^13^, ^14^ However, upon pooling these data, treatment effects became significant across several parameters of LV structure and function, as detailed above. It is likely that on an individual level, these studies lacked sufficient power to detect reverse cardiac remodelling and thus with pooling, this effect became evident.

Additionally, a trend towards improvement in GLS and LAVi was noted in our pooled analysis; however, neither of these parameters reached statistical significance. With respect to GLS, it is possible that the lack of treatment effect may be attributable to under‐powering, as the overall number of patients who had this parameter assessed was low (*n* = 327). This does not however explain the lack of significance in treatment effect on LAVi, given the population number was similar (*n* = 504) to other positive parameters such as LVEDV (*n* = 503). The heterogeneity of imaging modality could possibly have influenced outcome, as one would expect a higher sensitivity to changes in LAV with cardiac MRI.^20^ Interestingly, we did not find evidence of this upon sub‐group analysis of imaging modality utilized. An additional factor may be the low number of HFpEF patients in our cohort. We know that negative atrial remodelling is seen across the spectrum of HF phenotypes; however, there is some evidence to suggest that LA dysfunction may be more closely associated with poor outcomes in HFpEF.^21^Therefore, with a larger HFpEF population, it is plausible that this parameter may be of more relevance when evaluating treatment effects.

Previous data show that reverse cardiac remodelling is prognostically favourable in patients with HF and is associated with improved symptom scores and reduced mortality.^22^, ^23^, ^24^ This may, in part, explain the favourable clinical effects noted with SGLT2 inhibition in HF. One must note; however, the positive clinical effects of SGLT2 inhibition were noted very early in landmark studies, arguably before reverse remodelling could occur.^25^ Therefore, it is plausible that this is only a part of a very complex mechanistic puzzle. Treatment effects can also be influenced by population characteristics; therefore, it is important to note the demographics of our pooled‐population were largely reflective of the landmark trials such a DAPA HF and EMPEROR‐reduced, with approximately similar age, gender, NYHA class and percentage of diabetic patients included.^2^, ^26^

Insights into possible mechanisms for reverse cardiac remodelling may be gleaned from recent trials with data suggesting SGLT2 inhibition produces favourable changes in cardiac loading.^27^ Indeed, it has been postulated that these effects may be mediated by reduction of systemic blood pressure.^25^ However, data from EMPA‐HEART *(Effect of Empagliflozin on Left Ventricular Mass in Patients With Type 2 Diabetes Mellitus and Coronary Artery Disease*), which demonstrated significant improvements in LVM following SGLT2 inhibition, found that these changes were independent of systemic blood pressure changes.^28^ Additionally, the hypothesis that osmotic and diuretic effects mediate these changes, is not supported in the latest data from large, randomized trials. Indeed, the data show that diuretic effects are short lived and not corroborated by changes in other markers of volume.^29^, ^30^, ^31^ Furthermore, there is increasing evidence that SGLT2 inhibition directly inhibits pro‐inflammatory pathways responsible for cardiac fibrosis and adverse cardiac remodelling in HF and indeed this is borne out in several cardiac MRI studies.^28^, ^32^ Although positive end‐effects on cardiac remodelling are evident from this analysis, the mechanisms underpinning these changes remain uncertain, and further research is required.

The results of this study must be interpreted within the context of its limitations. First, the overall number of patients pooled are relatively low with substantial heterogeneity noted within the studies included. Second, the low sample size limits the assessment of publication bias given the reduction in power with a smaller number of included studies. Third, over half the studies included had a treatment duration of 1 year. Given that changes in cardiac structure may plausibly take longer to occur, a longer treatment duration may confer a more potent benefit of markers of reverse cardiac remodelling. Fourth, it is unclear if the remodelling benefit is influenced by diabetic status. Although the majority trials contained a mix of diabetic and non‐diabetic patients, final analysis of change in each respective cardiac parameters were often not sub‐categorized by diabetic status, we are therefore unable to ascertain the degree of interaction of this parameter on the final outcomes. Additionally, most patients included had HFrEF, with a limited number of studies in HFpEF. This may limit the applicability of results across the spectrum of LV impairment. Finally, within the trials included, only empagliflozin and dapagliflozin were included, it is therefore unclear from our analysis whether the beneficial effects extend to other available SGLT2 inhibitors.

## Conclusion

SGLT2 inhibitors have been demonstrated to reduced CV mortality and rates of HF hospitalization in patients with HF. Much interest surrounds the underlying mechanisms for these benefits and whether they are end results of synergy of systemic effects, direct cardiac effect or indeed both. This meta‐analysis of placebo‐controlled randomized trials in patients with HF demonstrates that treatment with SGLT2 inhibitors produces significant improvements in a variety of markers of reverse cardiac remodelling. These data add to the growing body of evidence that SGLT2 inhibition in patients with HF may have favourable direct effects on cardiac structure. More research is needed to evaluate the mechanisms underpinning these effects.

## Conflict of interest statement

Patrick Savage has no conflicts of interest to declare, Chris Watson has no conflicts of interest to declare, Brian Cox has no conflicts of interest to declare, Jaimie Coburn has no conflicts of interest to declare, Michael Shahmohammadi has no conflicts of interest to declare, David Grieve has no conflicts of interest to declare and Lana Dixon has no conflicts of interest to declare.

## Supporting information


**Figure S1:** Risk of bias summary of review authors' judgements about each risk of bias item for each included study. Green represents low risk. Summary table generated using review manager V5.3.
**Figure S2:** Risk of bias graph demonstrating review authors' judgements about each risk of bias item presented as percentages across all included studies. Summary graph generated using Review Manager V5.3.
**Supplemental Figure S3:** Publication Bias assessment.
**Figure S4:** Funnel plot visually demonstrating risk of publication bias for studies including LVESV (mls). Generated using JASP statistics software 0.13.3.
**Figure S5:** Funnel plot demonstrating risk of publication bias for studies including LVEDV (mls). Generated using JASP statistics software 0.13.3.
**Figure S6:** Funnel plot visually demonstrating risk of publication bias for studies including LVEF (%). Generated using JASP statistics software 0.13.3.
**Figure S7:** Funnel plot visually demonstrating risk of publication bias for studies including LV GLS (%). Generated using JASP statistics software 0.13.3.
**Figure S8:** Funnel plot visually demonstrating risk of publication bias for studies including LVM (g). Generated using JASP statistics software 0.13.3.
**Figure S9:** Funnel plot visually demonstrating risk of publication bias for studies including LVESVi (mls/m^2^). Generated using JASP statistics software 0.13.3.
**Figure S10:** Funnel plot visually demonstrating risk of publication bias for studies including LVEDVi (mls/m^2^). Generated using JASP statistics software 0.13.3.
**Figure S11:** Funnel plot visually demonstrating risk of publication bias for studies including LMi (g/m^2^). Generated using JASP statistics software 0.13.3.
