# Supplementary material for: Impact of SGLT2 inhibition on markers of reverse cardiac remodelling in heart failure: Systematic review and meta‐analysis
Source: ESC Heart Fail. 2024 Jul 26;11(6):3636–48. doi: 10.1002/ehf2.14993 (PMC11631341; doi:10.1002/ehf2.14993)
Supplement: Supplementary file 1 — Figure S1: Risk of bias summary of review authors' judgements about each risk of bias item for each included study. Green represents low risk. Summary table generated using review manager V5.3. Figure S2: Risk of bias graph demonstrating review authors' judgements about each risk of bias item presented as percentages across all included studies. Summary graph generated using Review Manager V5.3. Supplemental Figure S3: Publication Bias assessment. Figure S4: Funnel plot visually demonstrating risk of publication bias for studies including LVESV (mls). Generated using JASP statistics software 0.13.3. Figure S5: Funnel plot demonstrating risk of publication bias for studies including LVEDV (mls). Generated using JASP statistics software 0.13.3. Figure S6: Funnel plot visually demonstrating risk of publication bias for studies including LVEF (%). Generated using JASP statistics software 0.13.3. Figure S7: Funnel plot visually demonstrating risk of publication bias for studies including LV GLS (%). Generated using JASP statistics software 0.13.3. Figure S8: Funnel plot visually demonstrating risk of publication bias for studies including LVM (g). Generated using JASP statistics software 0.13.3. Figure S9: Funnel plot visually demonstrating risk of publication bias for studies including LVESVi (mls/m2). Generated using JASP statistics software 0.13.3. Figure S10: Funnel plot visually demonstrating risk of publication bias for studies including LVEDVi (mls/m2). Generated using JASP statistics software 0.13.3. Figure S11: Funnel plot visually demonstrating risk of publication bias for studies including LMi (g/m2). Generated using JASP statistics software 0.13.3. [file EHF2-11-3636-s001.docx]

**APPENDIX**

**1.0. Protocol**

**1.1. Objective**

The primary objective of this study is to conduct a systematic review and meta-analysis of randomised placebo-controlled trials comparing the effect of SGLT2 inhibition on cardiac remodeling parameters in patients with heart failure.

**1.2. Methods**

A systematic review and meta-analysis will be conducted in accordance with the Preferred Reporting Items for Systematic Review and Meta-Analysis (PRISMA) Statement and Cochrane Collaboration. In this systemic review, we will retrieve the studies that compare the effects of SGLT2 inhibitors (empagliflozin, canagliflozin, dapagliflozin, ertugliflozin, ipragliflozin, luseogliflozin, remogliflozin, sotagliflozin, and licogliflozin) on cardiac remodelling parameters evaluated using echocardiography or cardiac magnetic resonance imaging in patients with heart failure. This study has been registered at ***PROSPERO*** with registration number **CRD42023482722**.

**1.3. Data Sources and Search strategies**

We will search the PubMed, EMBASE, MEDLINE and Cochrane Library databases published in English from inception up to January 7^th^, 2024, for RCTs on SGLT2 inhibitor treatment in patients with HF. The literature search will be designed by an information specialist utilizing selected medical subject heading (MeSH) terms and free-text terms associated with SGLT2 inhibitors and LV remodeling. We will also screen the references of the included studies, reviews, and meta-analyses to identify any additional trials.

**1.4. Screening criteria**

- ***Population:*** We will include patients >18 years of age, of both male and female gender, diagnosed with either heart failure with reduced ejection fraction (HFreF) or heart failure with preserved ejection fraction (HFpEF).
- ***Intervention:*** We will include studies evaluate SGLT2 inhibitors including empagliflozin, canagliflozin, dapagliflozin, ertugliflozin, ipragliflozin, luseogliflozin, remogliflozin, Sotagliflozin and licogliflozin.
- ***Comparison:*** We will include randomised controlled trials comparing the effects of SGLT2 inhibition vs placebo on markers of left ventricular remodeling as evaluated using cardiac imaging with either echocardiography or cardiac magnetic resonance imaging.
- ***Outcomes:*** Left ventricular end diastolic volume [LVEDV (mls)], left ventricular end systolic volume [LVESV (mls)], Left ventricular mass [LVM (g)], left ventricular end diastolic volume and volume index [LVEDVi (mls/m2)], left ventricular end systolic volume and volume index [LVSDVi (mls/m2)], left ventricular ejection fraction [LVEF) (%)], left ventricular mass index [LVMi] (g/m2)], left atrial volume index [LAVi (mls/m2)] and left ventricular global longitudinal strain (%).

**1.5. Study selection**

We will include randomised controlled trials published in English. Observational and cohort studies will be excluded. Case reports, series, editorial, letters and perspectives will be excluded. Existing meta-analyses and systematic reviews will be excluded. Databases utilized for search will include PubMed, Embase, Cochrane and MEDLINE.

**1.6. Study selection and data extraction**

Two reviewers (BC and MS) will independently screen titles and abstracts for eligible studies after the literature search. Further full text evaluation will be performed if appraisal of the title is not sufficient to decide on inclusion. A third reviewer (PS) will mediate and solve any discrepancies in eligibility. Data extraction of eligible studies will then be performed by the two reviewers (BC and MS). The data will include general information, population and settings, methods, participants, interventions, outcomes and results. Outcome measurements will be extracted as continuous or dichotomous.

**1.7. Risk of bias assessment**

Risk of bias of the included studies will be assessed using a tool developed by the Cochrane Collaboration. This tool includes six domains: random sequence generation, allocation concealment, blinding, incomplete outcome data, selective outcome reporting and other bias. The six domains will be separately evaluated and categorised as low, unclear or high risk of bias.

**1.8. Data analysis**

The results will quantitatively combined and analysed using Review Manager (RevMan) using general approaches according to the Cochrane Handbook. In studies without standard deviations, p-values, and confidence intervals (CIs), the square root of weighted mean variance of all other studies was used to estimate the standard deviation. In studies without mean and standard deviations, the median and interquartile range will be used to estimate mean and standard deviations. In studies without standard deviation, the p-value or CI was used to calculate the standard deviations.

**1.9. Missing data**

Scenarios of missing data are commonly encountered in data extraction. We will contact authors by email to ask for original data.

**1.10. Sensitivity analysis**

We will first exclude trials with high risk of bias to check if the results are consistent. Then we will exclude trials that are not published in peer-review journals to examine whether the data source influences the results.

**2.0. Search strategy**

We searched the PubMed, EMBASE, MEDLINE and Cochrane Library databases published in English from inception up to January 7^th^, 2024, for RCTs on SGLT2 inhibitor treatment in patients with HF. The literature search was designed by an information specialist utilizing selected medical subject heading (MeSH) terms and free-text terms associated with SGLT2 inhibitors and LV remodeling. We will also screen the references of the included studies, reviews, and meta-analyses to identify any additional trials. The search was restricted to studies published in English. The search strategies for each database are displayed below:

**2.1. MEDLINE (Ovid interface)**

| **#** | Query | Results from 7^th^ Jan 2024 |
| --- | --- | --- |
| 1 | Sodium-Glucose Transporter 2 Inhibitors/ | 6,002 |
| 2 | dapagliflozin.mp. | 2,197 |
| 3 | Canagliflozin/ | 1,044 |
| 4 | ertugliflozin.mp. | 201 |
| 5 | ipragliflozin.mp. | 229 |
| 6 | luseogliflozin.mp. | 122 |
| 7 | remogliflozin.mp. | 42 |
| 8 | Sotagliflozin.mp. | 154 |
| 9 | licogliflozin.mp. | 15 |
| 10 | Heart Failure/ | 146,256 |
| 11 | Randomized Controlled Trials as Topic/ | 165,091 |
| 12 | placebo.mp. | 228,245 |
| 13 | cardiac imaging techniques/ or echocardiography/ | 100,775 |
| 14 | Ventricular Remodeling/ | 14,956 |
| 15 | 1 or 2 or 3 or 4 or 5 or 6 or 7 or 8 or 9 | 7,450 |
| 16 | 10 and 15 | 1,378 |
| 17 | 11 or 12 | 368,143 |
| 18 | 15 and 16 and 17 | 393 |

**2.2. PUBMED database**

("echocardiographies"[All Fields] OR "echocardiography"[MeSH Terms] OR "echocardiography"[All Fields] OR ("echocardiographies"[All Fields] OR "echocardiography"[MeSH Terms] OR "echocardiography"[All Fields]) OR ("cardiac imaging techniques"[MeSH Terms] OR ("cardiac"[All Fields] AND "imaging"[All Fields] AND "techniques"[All Fields]) OR "cardiac imaging techniques"[All Fields]) OR ("ventricular remodelling"[All Fields] OR "ventricular remodeling"[MeSH Terms] OR ("ventricular"[All Fields] AND "remodeling"[All Fields]) OR "ventricular remodeling"[All Fields]) OR ("ventricular remodelling"[All Fields] OR "ventricular remodeling"[MeSH Terms] OR ("ventricular"[All Fields] AND "remodeling"[All Fields]) OR "ventricular remodeling"[All Fields]) OR ("placeboes"[All Fields] OR "placebos"[MeSH Terms] OR "placebos"[All Fields] OR "placebo"[All Fields])) AND ("randomized controlled trial"[Publication Type] OR "randomized controlled trials as topic"[MeSH Terms] OR "randomised controlled trials"[All Fields] OR "randomized controlled trials"[All Fields]) AND ("heart failure"[MeSH Terms] OR ("heart"[All Fields] AND "failure"[All Fields]) OR "heart failure"[All Fields]) AND ("sodium glucose transporter 2 inhibitors"[Pharmacological Action] OR "sodium glucose transporter 2 inhibitors"[MeSH Terms] OR "sodium glucose transporter 2 inhibitors"[All Fields] OR ("sglt2"[All Fields] AND "inhibitors"[All Fields]) OR "sglt2 inhibitors"[All Fields])

**2.3. Central Register of Cochrane controlled trials (CENTRAL)**

Date Run: 6/01/2024 11:53:00

ID Search Hits

#1 MeSH descriptor: [Sodium-Glucose Transporter 2 Inhibitors] explode all trees 777

#2 MeSH descriptor: [Heart Failure] explode all trees 14733

#3 MeSH descriptor: [Randomized Controlled Trial] explode all trees 25729

#4 MeSH descriptor: [Echocardiography] explode all trees 5490

#5 MeSH descriptor: [Magnetic Resonance Imaging] explode all trees 11024

#6 #2 AND #3 433

#7 #1 OR #6 1210

#8 #4 OR #5 16320

#9 #7 AND #8 62

**2.4. Ovid MEDLINE(R) ALL <1946 to January 7, 2024>**

1 Sodium-Glucose Transporter 2 Inhibitors/ 6197

2 DAPAGLIFLOZIN.mp. 2964

3 EMPAGLIFLOZIN.mp. 3051

4 Canagliflozin/ 1065

5 ertugliflozin.mp. 286

6 ipragliflozin.mp. 303

7 luseogliflozin.mp. 157

8 remogliflozin.mp. 50

9 Sotagliflozin.mp. 204

10 licogliflozin.mp. 15

11 1 or 2 or 3 or 4 or 5 or 6 or 7 or 8 or 9 or 10 9923

12 Heart Failure/ 148039

13 11 and 12 1655

14 placebo.mp. or Clinical Trials as Topic/ 438048

15 13 and 14 441

16 Cardiac Imaging Techniques/ 1913

17 14 or 16 439936

18 15 and 17 441

**3.0. Risk of bias assessments**

**3.1. Supplemental Figure 1: Risk of bias summary table**


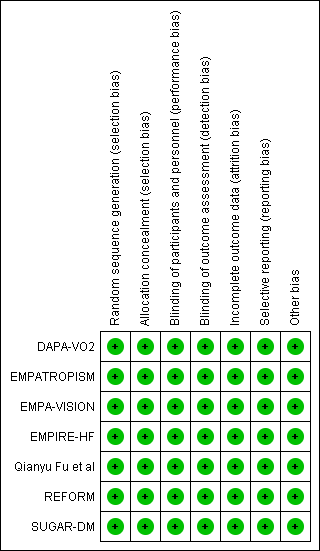


**Figure S1:** Risk of bias summary of review authors' judgements about each risk of bias item for each included study. Green represents low risk. Summary table generated using review manager V5.3.

**3.2. Supplemental Figure 2: Risk of bias graph**


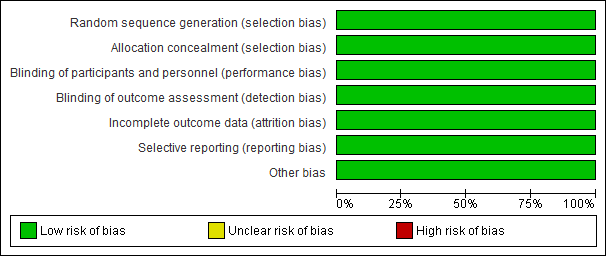


**Figure S2:** Risk of bias graph demonstrating review authors' judgements about each risk of bias item presented as percentages across all included studies. Summary graph generated using Review Manager V5.3.

**4.0. Assessment of publication Bias**

**4.1. Quantitative assessment of publication bias**

| **Supplemental Figure-S3: Publication Bias assessment** | | | | |
| --- | --- | --- | --- | --- |
|  | ***Nonparametric rank correlation test (Begg)*** | | **Regression-based Egger test** | |
|  | **Kendall's score** | ***P*** | ***z*** | ***P*** |
| **LVESV** | 0.000 | 1.000 | 1.035 | 0.300 |
| **LVEDV** | 0.200 | 0.719 | 0.994 | 0.320 |
| **LVEF** | -0.048 | 1.000 | -1.015 | 0.310 |
| **LV GLS** | 0.333 | 0.750 | 0.988 | 0.323 |
| **LVM** | -0.447 | 0.296 | -1.146 | 0.252 |
| **LVESVi** | -0.200 | 0.817 | 1.575 | 0.115 |
| **LVEDVi** | 0.400 | 0.483 | 2.367 | 0.018 |
| **LAVi** | -0.200 | 0.817 | 0.381 | 0.703 |
| **LVMi** | -0.067 | 1.000 | -0463 | 0.644 |

**4.2. Funnel plots demonstrating visual assessment of publication bias**

**Supplemental Figure 4: *Left ventricular end-systolic volume (LVESV) (mls)***

***
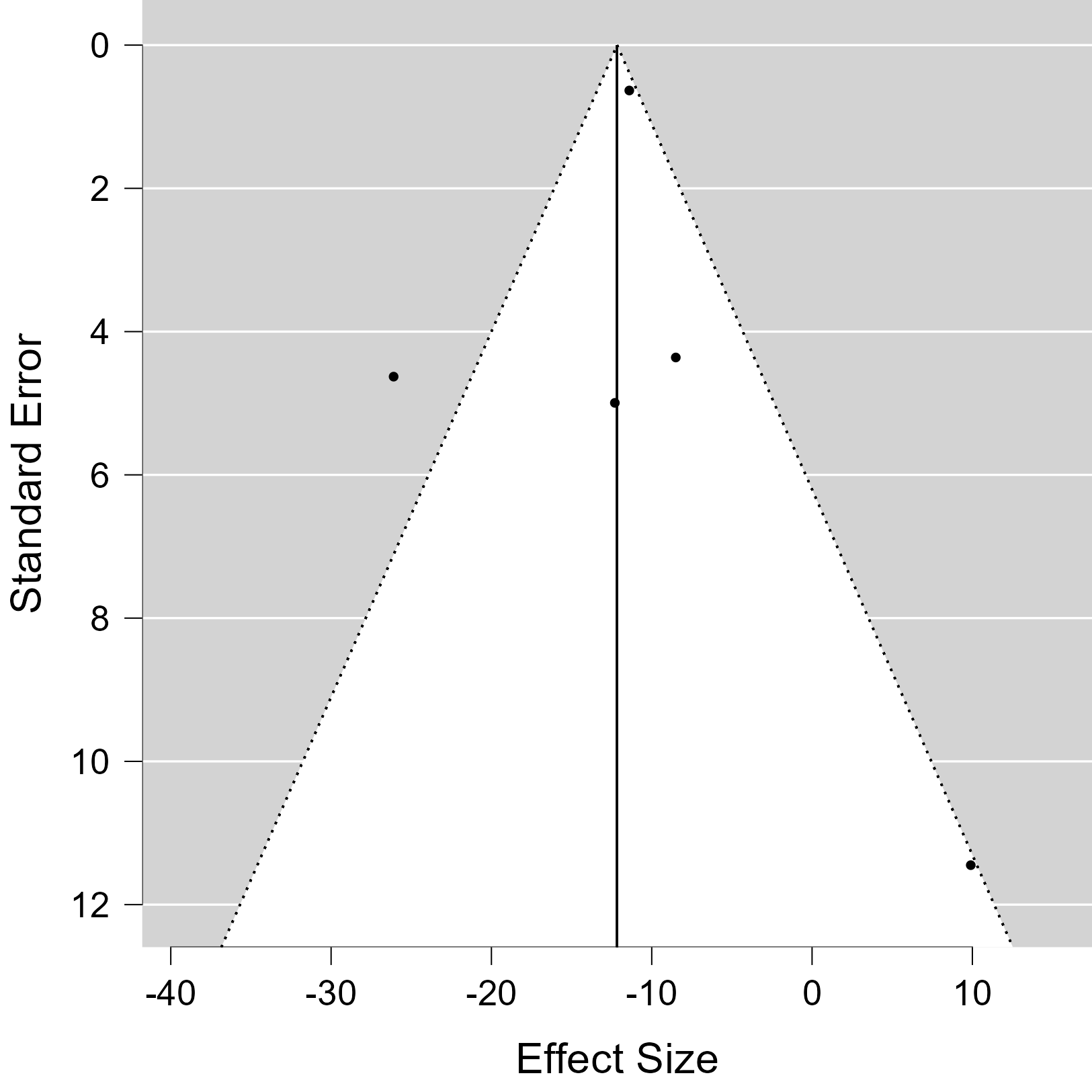
***

**Figure S4:** Funnel plot visually demonstrating risk of publication bias for studies including LVESV (mls). Generated using JASP statistics software 0.13.3.

**Supplemental Figure 5: *Left ventricular end-systolic volume (LVEDV) (mls)***

**
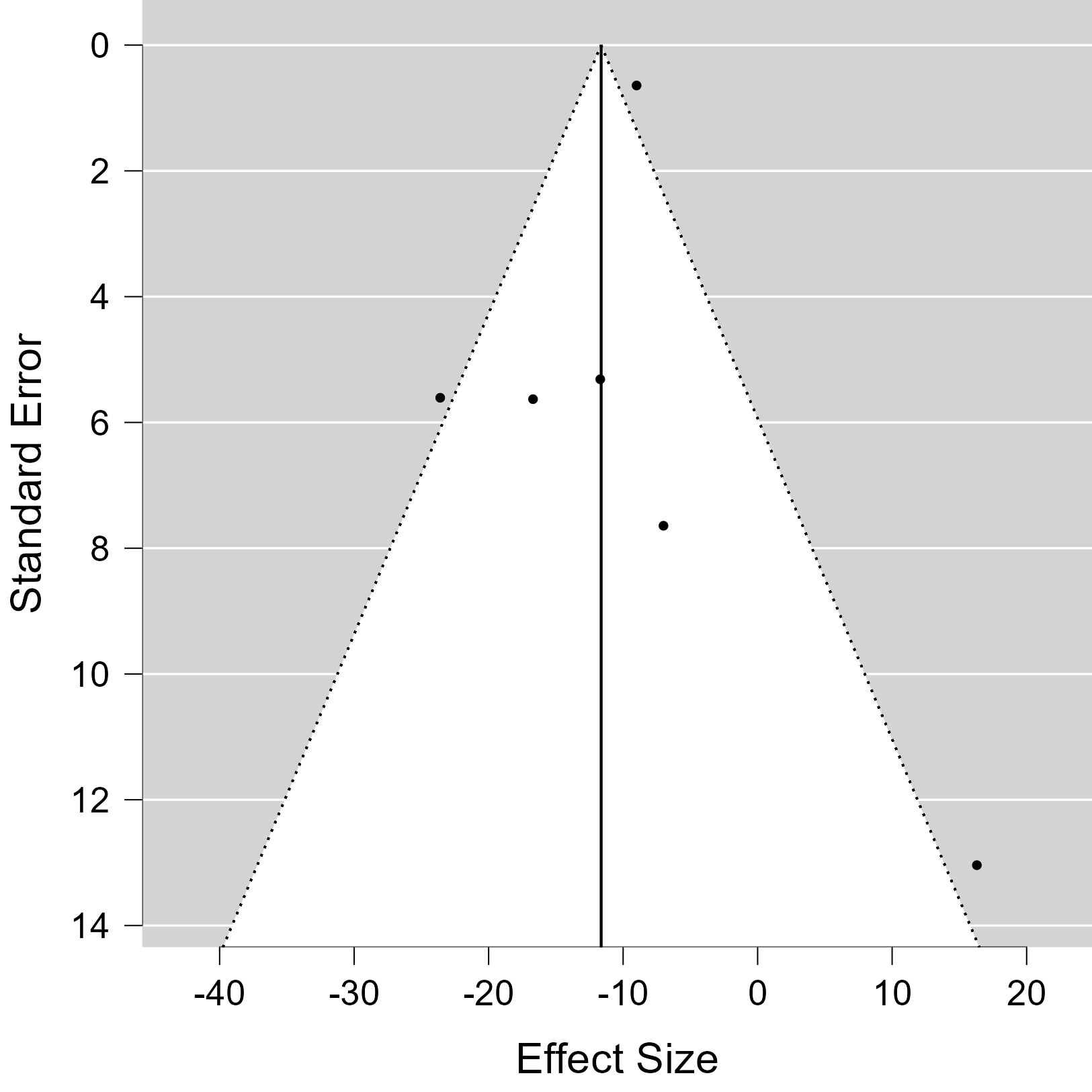
**

**Figure S5:** Funnel plot demonstrating risk of publication bias for studies including LVEDV (mls). Generated using JASP statistics software 0.13.3.

**Supplemental Figure 6: *Left ventricular ejection fraction (%)***

***
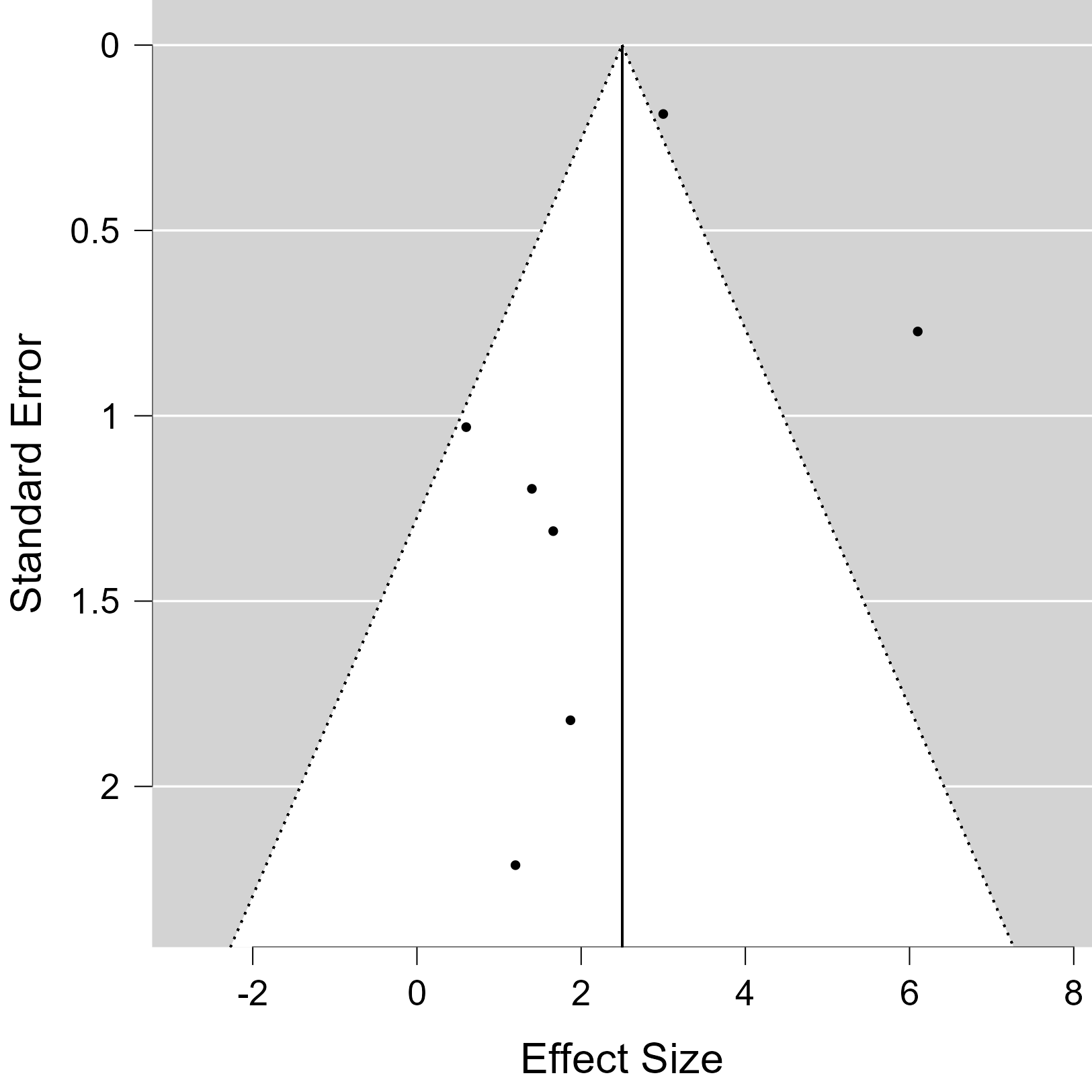
***

**Figure S6:** Funnel plot visually demonstrating risk of publication bias for studies including LVEF (%). Generated using JASP statistics software 0.13.3.

**Supplemental Figure 7: *Left ventricular global longitudinal strain (%)***

***
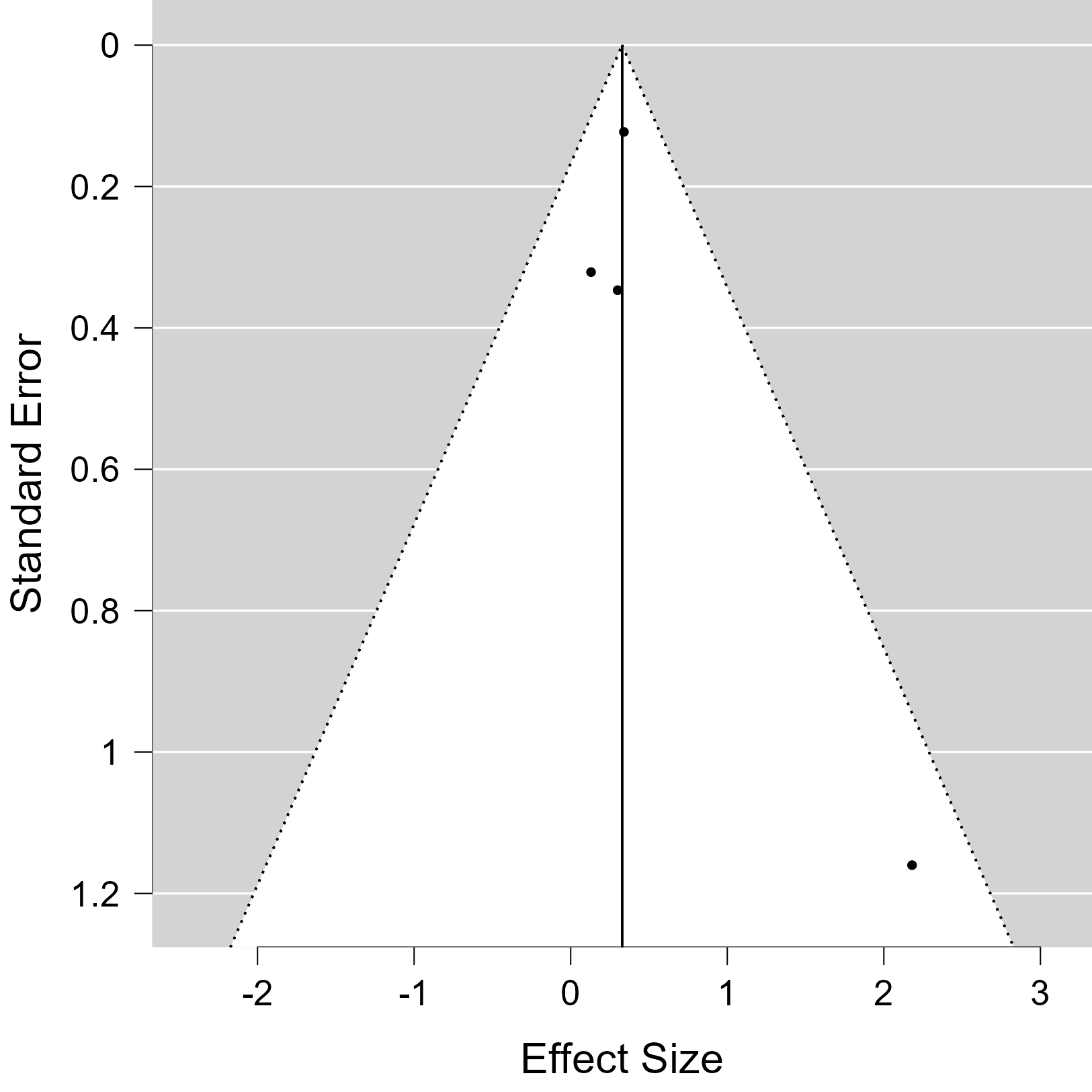
***

**Figure S7:** Funnel plot visually demonstrating risk of publication bias for studies including LV GLS (%). Generated using JASP statistics software 0.13.3.

**Supplemental Figure 8: *Left ventricular Mass [LVM (g)]***


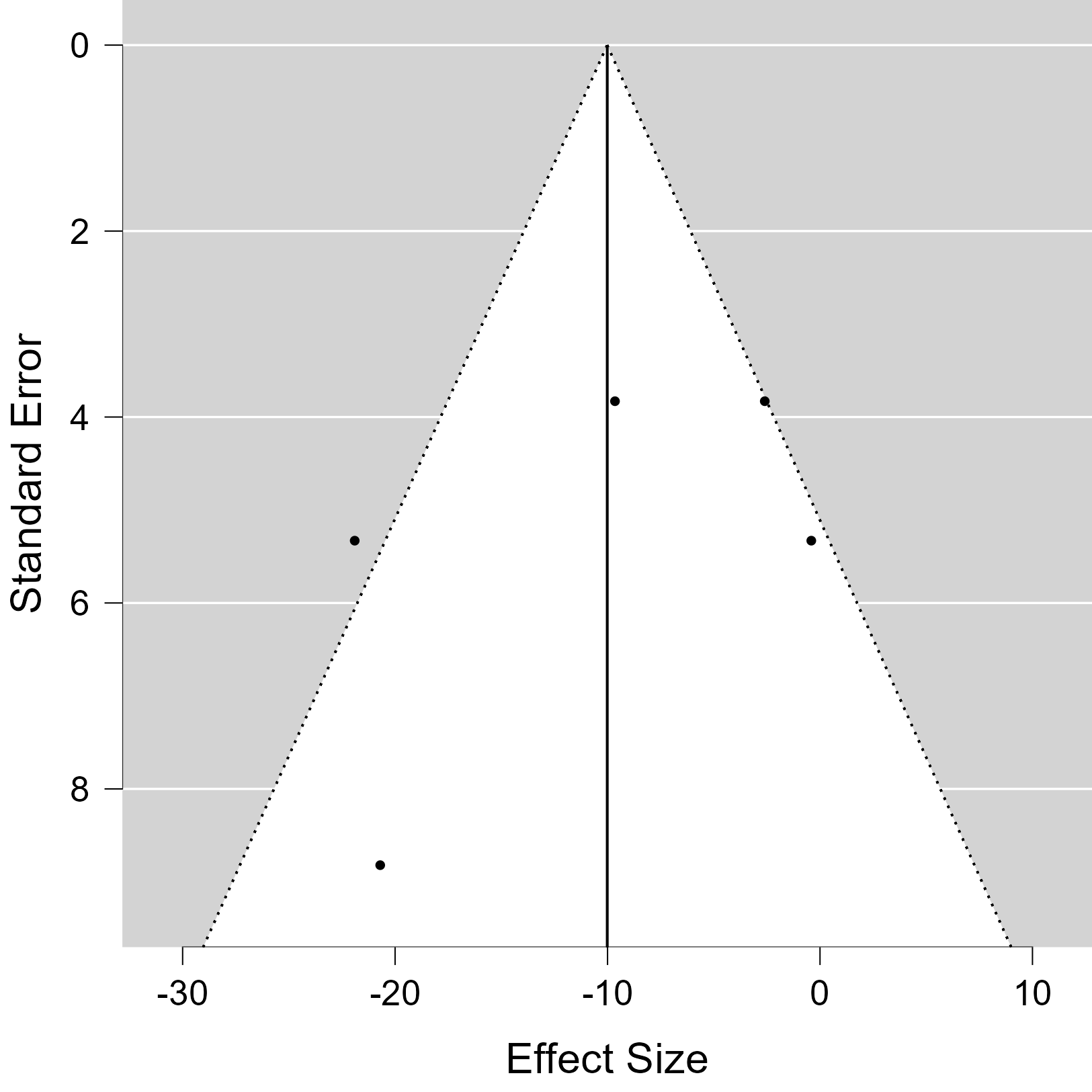


**Figure S8:** Funnel plot visually demonstrating risk of publication bias for studies including LVM (g). Generated using JASP statistics software 0.13.3.

**Supplemental Figure 9: Left ventricular end-systolic volume index [*LVESVi )mls/m^2^)]***

***
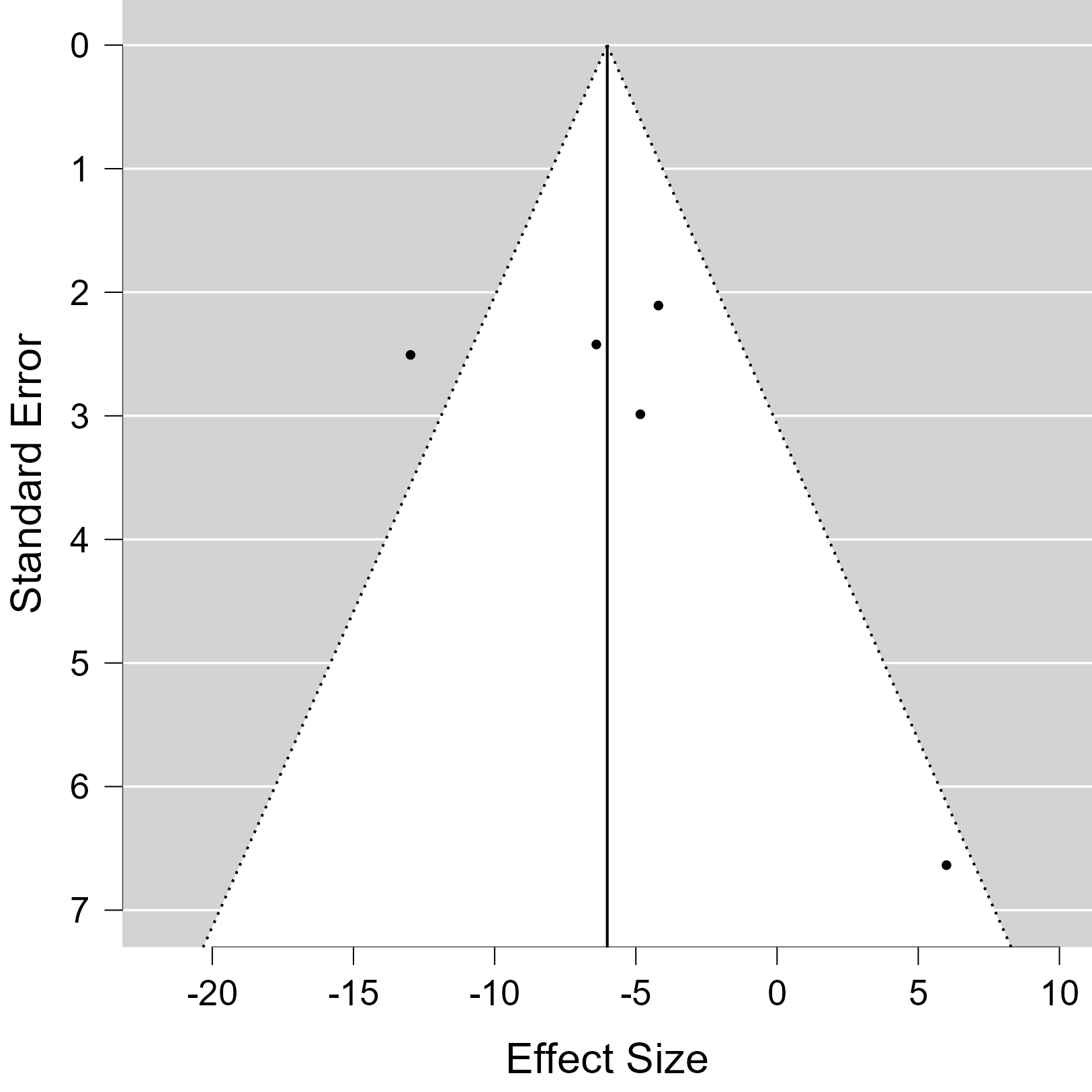
***

**Figure S9:** Funnel plot visually demonstrating risk of publication bias for studies including LVESVi (mls/m^2^). Generated using JASP statistics software 0.13.3.

**Supplemental Figure 10: *Left ventricular end-diastolic volume index [LVEDVi (mls/m^2^)]***

***
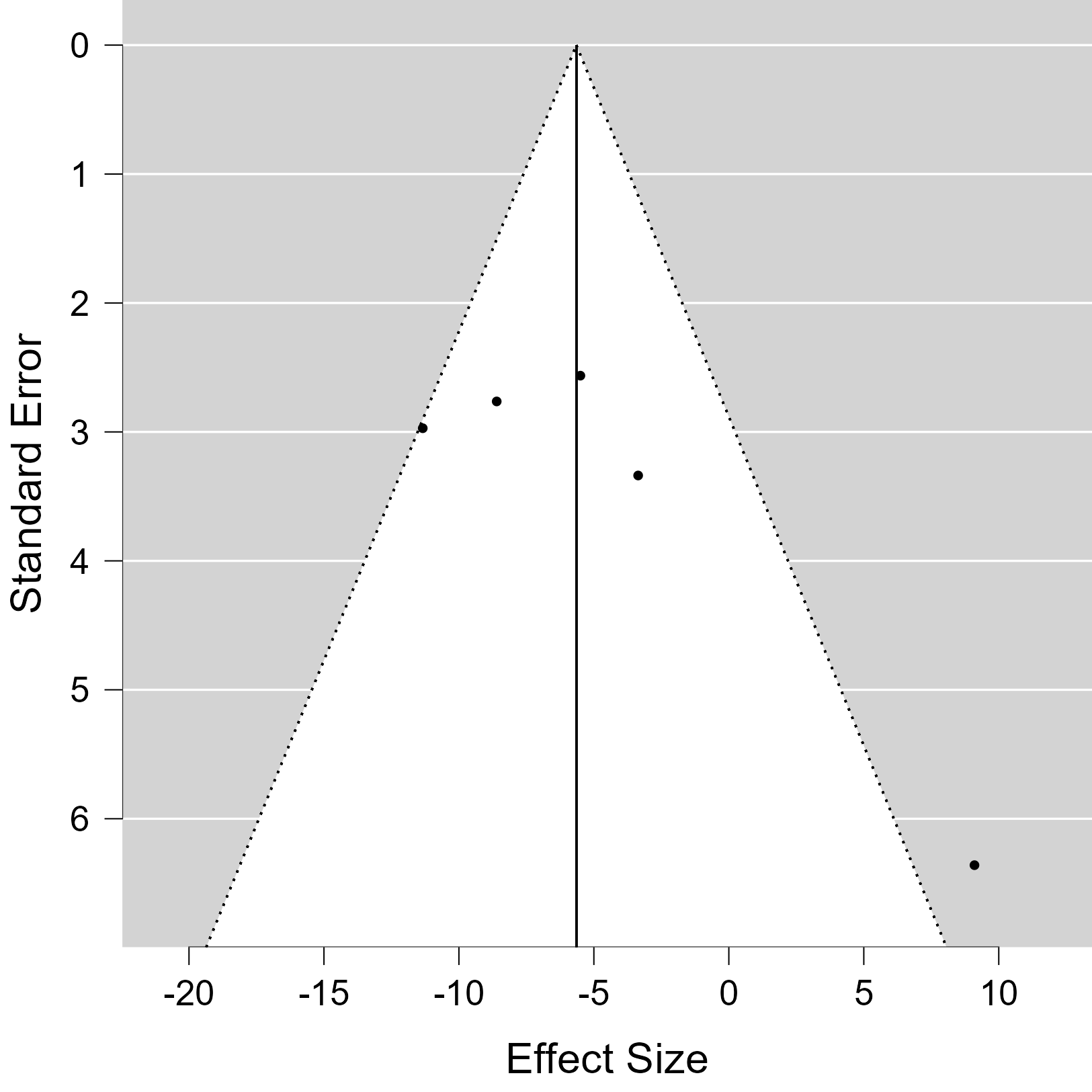
***

**Figure S10:** Funnel plot visually demonstrating risk of publication bias for studies including LVEDVi (mls/m^2^). Generated using JASP statistics software 0.13.3.

**Supplemental Figure 8: Left atrial volume index [LAVi (mls/m^2^)]**

***
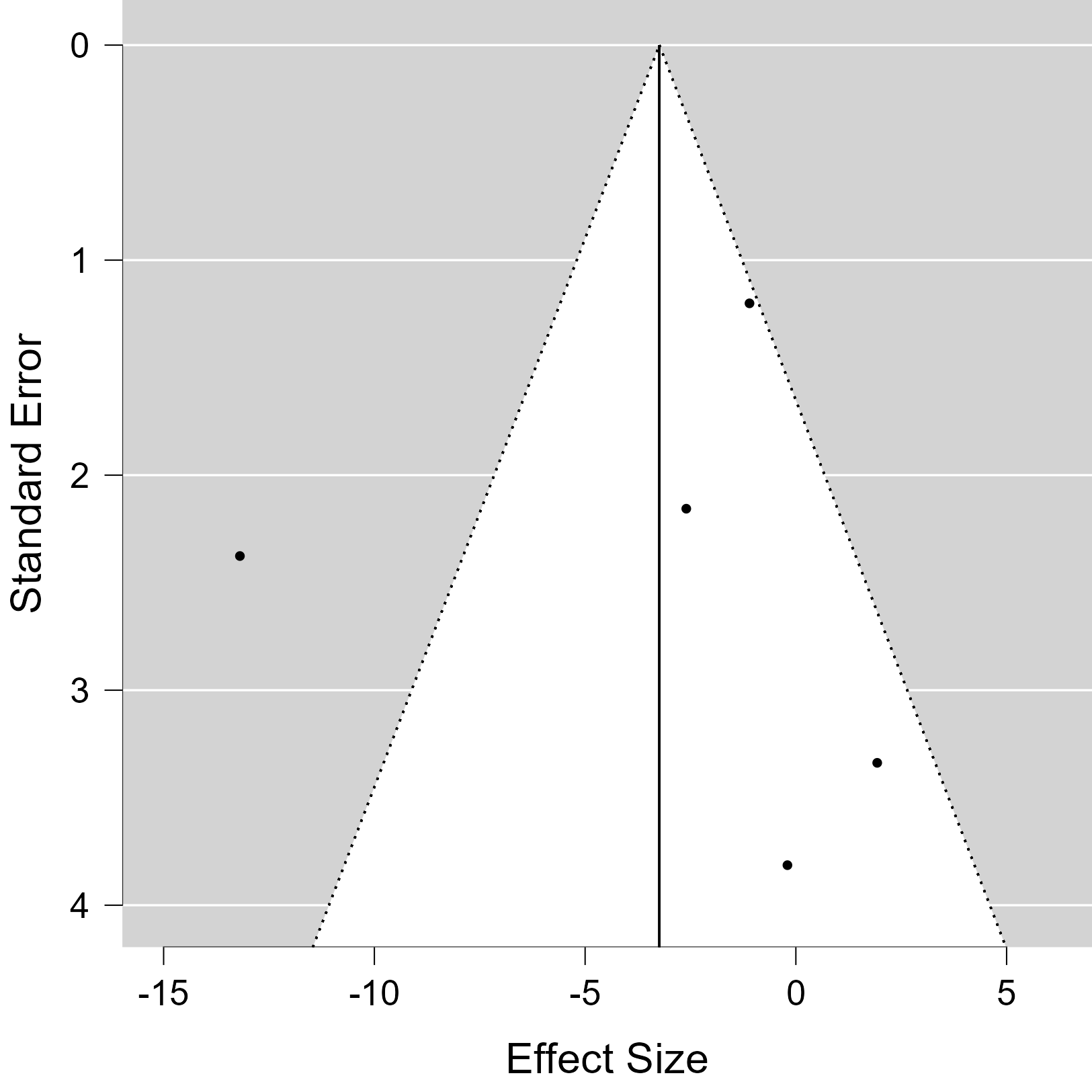
***

**Figure S10:** Funnel plot visually demonstrating risk of publication bias for studies including LAVi (mls/m^2^). Generated using JASP statistics software 0.13.3.

**Supplemental Figure 11: LV Mass index [LVMi (g/m^2^)]**


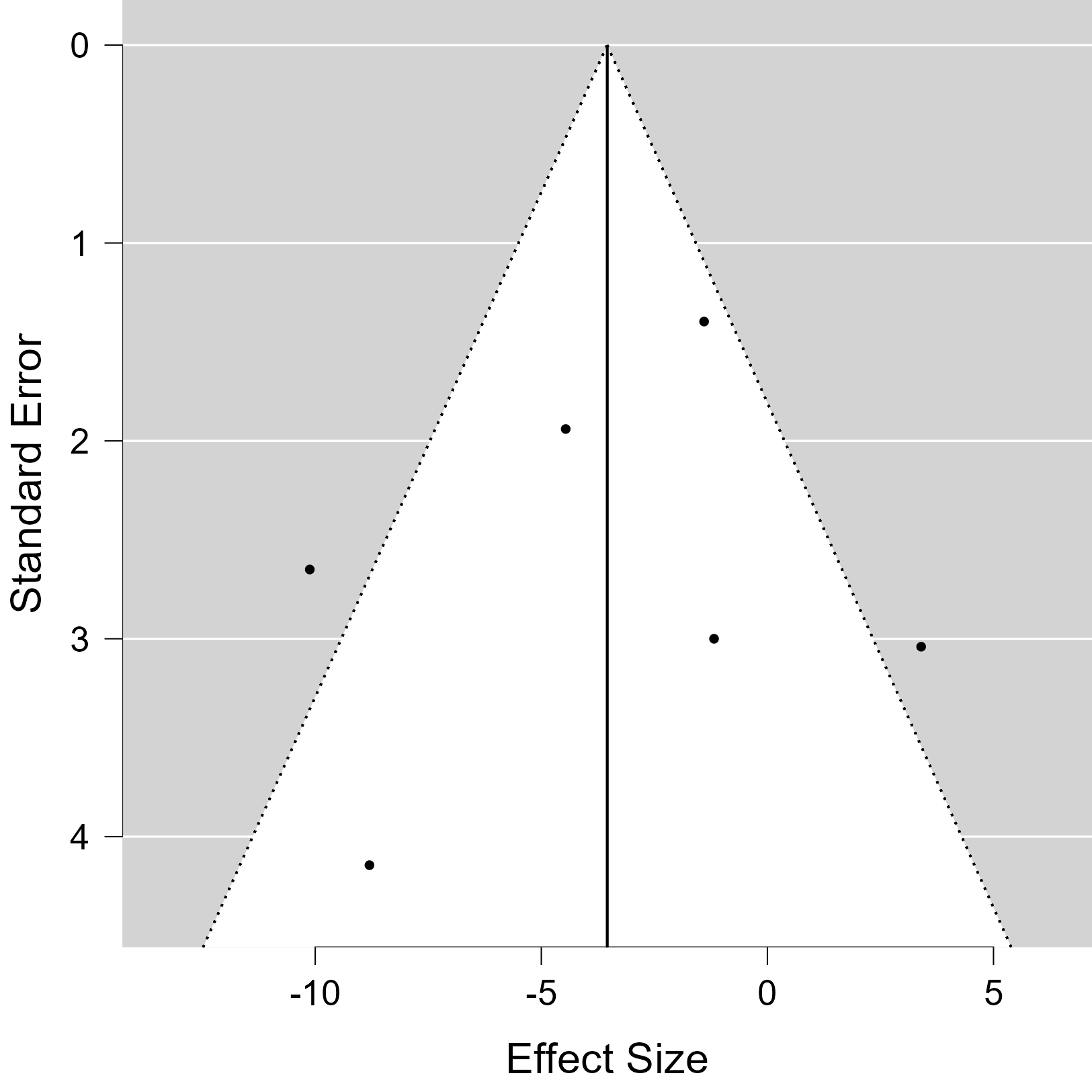


**Figure S11:** Funnel plot visually demonstrating risk of publication bias for studies including LMi (g/m^2^). Generated using JASP statistics software 0.13.3.

.

**5.0. Sensitivity analysis utilising standardised mean difference (SMD) for all assessed parameters.**

**5.1. LVESV (mls)**

**
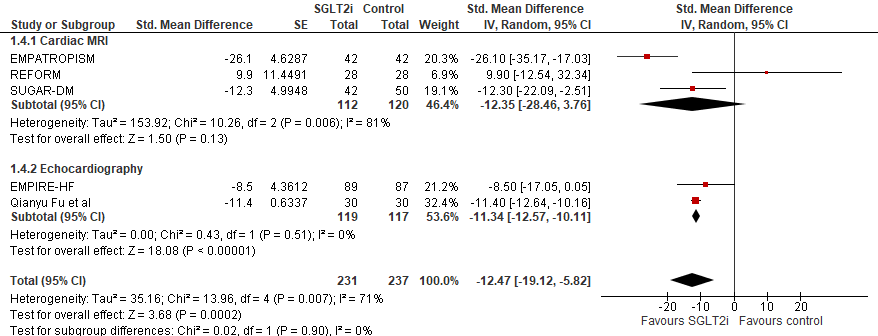
**

**5.2. LVESVi (mls/m^2^)**

**
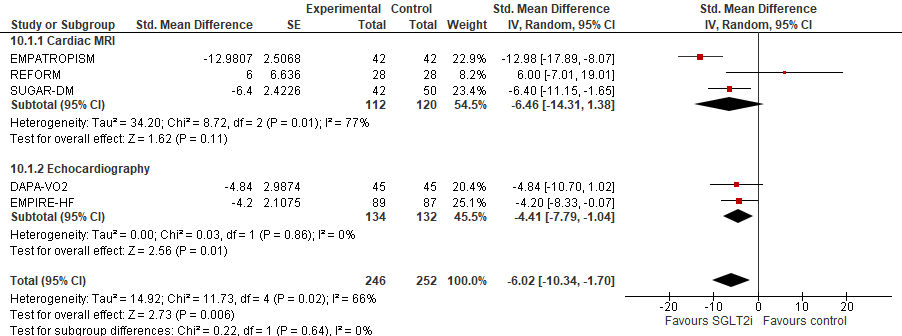
**

**5.3. LVEDV (mls)**

**
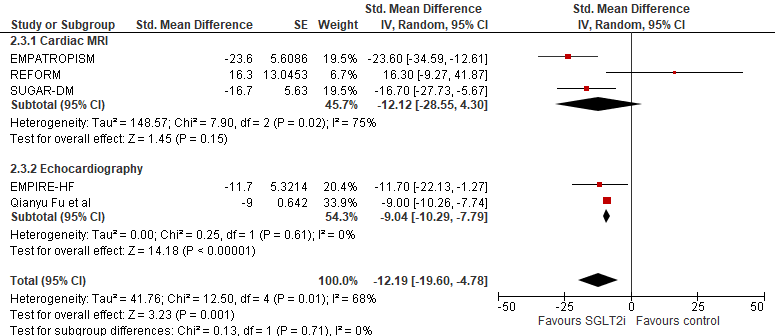
**

**5.4. LVESDi (mls/m^2^)**

**
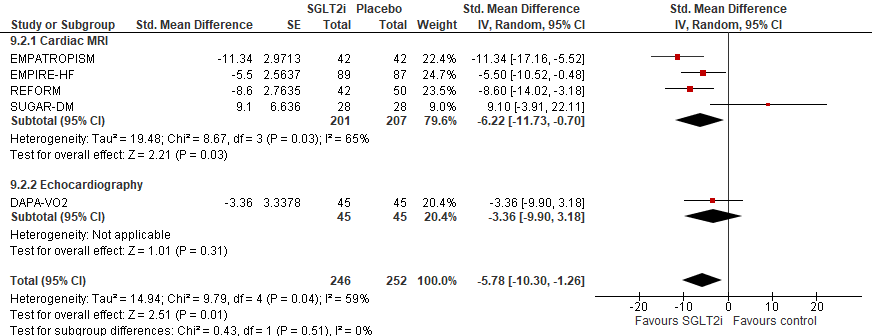
**

**5.5. LAVi (mls/m^2^)**

**
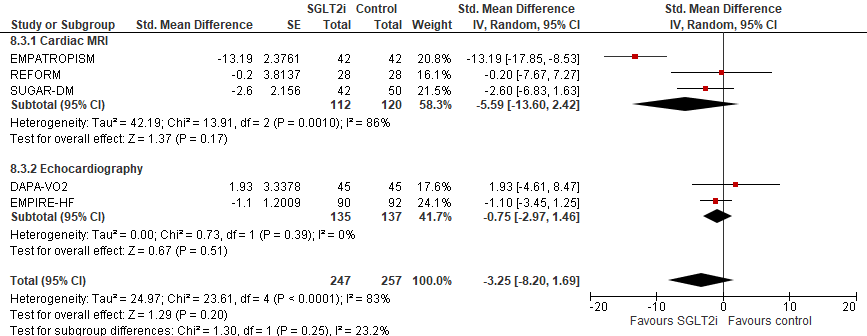
**

**5.6. LVM (g)**

**
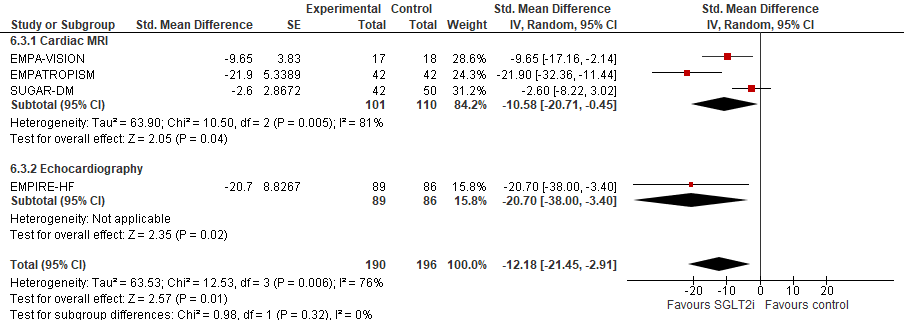
**

**5.7. LVMi (g/m^2^)**

**
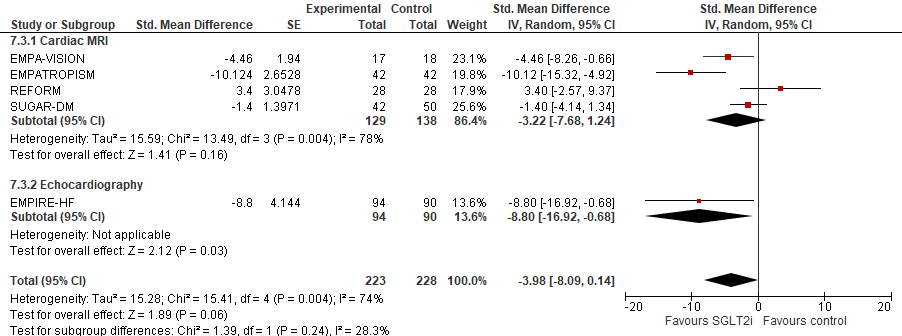
**

**5.8. LVEF (%)**

**
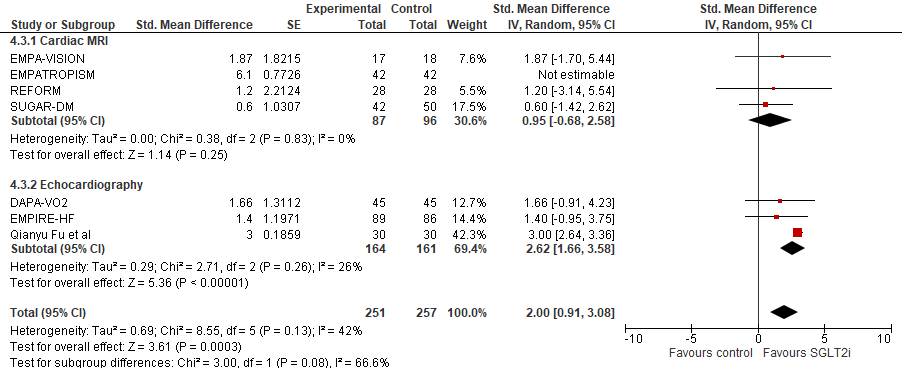
**

**5.9. LV GLS (%)**

**
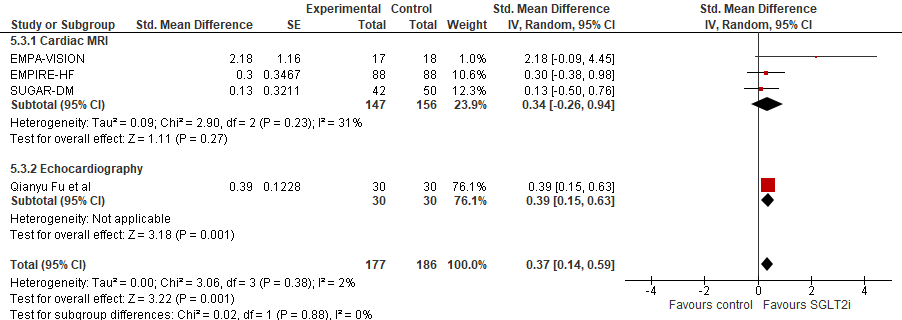
**

**6.0. Sensitivity analysis excluding REFORM study**

**6.1. LVESV (mls)**


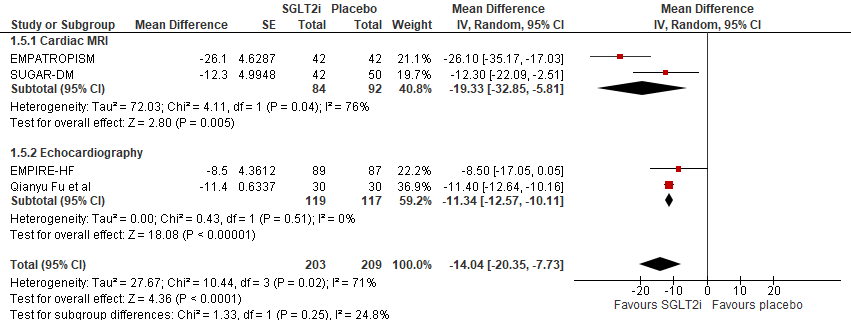


**6.1:** Forrest plot demonstrating mean difference in LVESV (mls) baseline to study end in RCTs of patients with heart failure treated with SGLT2 inhibitor therapy versus controls with REFORM trial excluded. Overall heterogeneity did not change after exclusion of REFORM trial (I^2^=71% to 71%) however, overall change in LVESV was more pronounced (mean difference -12.47mls to -14.04mls; p<0.0001). CI: Confidence interval, SE: Standard error.

**6.2. LVESVi (mls/m^2^)**


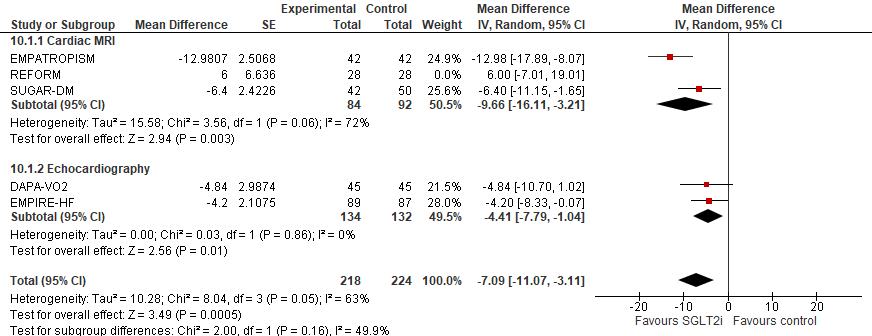


**6.2:** Forrest plot demonstrating mean difference in LVESVi (mls/m^2^) baseline to study end in RCTs of patients with heart failure treated with SGLT2 inhibitor therapy versus controls with REFORM trial excluded. Overall heterogeneity fell slightly from I^2^=66% to 63%. Overall change in LVESVi was more pronounced (mean difference -6.02mls to -7.09mls) and remained significant p<0.0005).

CI: Confidence interval, SE: Standard error.

**6.3. LVEDV (mls)**


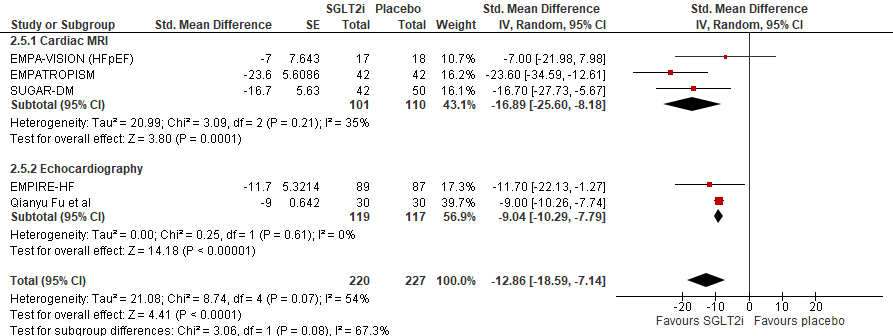


**6.3:** Forrest plot demonstrating mean difference in LVEDV (mls) baseline to study end in RCTs of patients with heart failure treated with SGLT2 inhibitor therapy versus controls with REFORM trial excluded. Overall heterogeneity fell slightly from I^2^=60% to 54%. Overall change in LVEDV was more pronounced (mean difference -11.62mls to -12.86mls) and remained significant p<0.0001).

CI: Confidence interval, SE: Standard error.

**6.4. LVEDVi (mls/m^2^)**


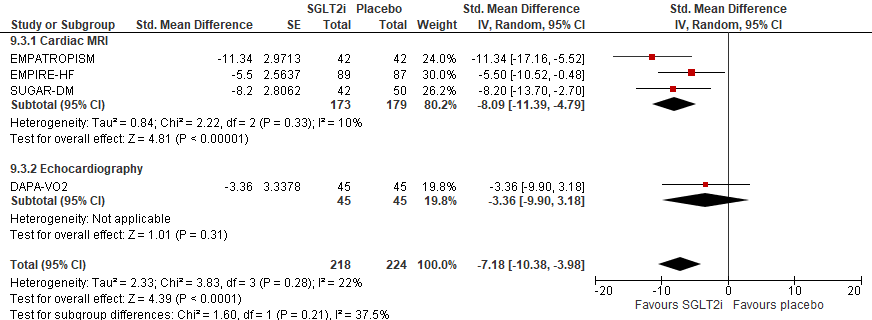


**6.4:** Forrest plot demonstrating mean difference in LVEDVi (mls/m^2^) baseline to study end in RCTs of patients with heart failure treated with SGLT2 inhibitor therapy versus controls with REFORM trial excluded. Overall heterogeneity fell significantly from I^2^=47% to 22%. Overall change in LVEDVi was more pronounced (mean difference -6.08mls to -7.18mls) and remained significant p<0.0001).

CI: Confidence interval, SE: Standard error.

**6.5. LVMi (g/m^2^)**


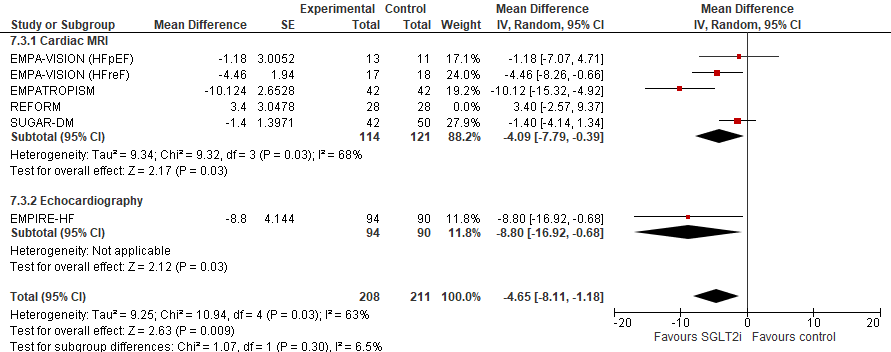


**6.5:** Forrest plot demonstrating mean difference in LVMi (g/m^2^) baseline to study end in RCTs of patients with heart failure treated with SGLT2 inhibitor therapy versus controls with REFORM trial excluded. Overall heterogeneity did not change I^2^=63% to 63%. Overall change in LVMi was not altered (mean difference -4.65mls to -4.65mls) and remained significant p<0.009).

CI: Confidence interval, SE: Standard error.
